# Comparative in depth RNA sequencing of *P*. *tricornutum*’s morphotypes reveals specific features of the oval morphotype

**DOI:** 10.1038/s41598-018-32519-7

**Published:** 2018-09-25

**Authors:** Clément Ovide, Marie-Christine Kiefer-Meyer, Caroline Bérard, Nicolas Vergne, Thierry Lecroq, Carole Plasson, Carole Burel, Sophie Bernard, Azeddine Driouich, Patrice Lerouge, Isabelle Tournier, Hélène Dauchel, Muriel Bardor

**Affiliations:** 1 Normandie Univ, UNIROUEN, Laboratoire Glyco-MEV EA4358, 76000 Rouen, France; 2 Normandie Univ, UNIROUEN, LITIS EA 4108, 76000 Rouen, France; 3 Normandie Univ, UNIROUEN, LMRS UMR 6085 CNRS, 76000 Rouen, France; 4 Normandie Univ, UNIROUEN, Plate-forme PRIMACEN, 76000 Rouen, France; 5 Normandie Univ, UNIROUEN, Inserm U1079, IRIB Genomic Facility, 76000 Rouen, France; 6 Institut Universitaire de France (IUF), Paris, France

## Abstract

*Phaeodactylum tricornutum* is the most studied diatom encountered principally in coastal unstable environments. It has been hypothesized that the great adaptability of *P*. *tricornutum* is probably due to its pleomorphism. Indeed, *P*. *tricornutum* is an atypical diatom since it can display three morphotypes: fusiform, triradiate and oval. Currently, little information is available regarding the physiological significance of this morphogenesis. In this study, we adapted *P*. *tricornutum* Pt3 strain to obtain algal culture particularly enriched in one dominant morphotype: fusiform, triradiate or oval. These cultures were used to run high-throughput RNA-Sequencing. The whole mRNA transcriptome of each morphotype was determined. Pairwise comparisons highlighted biological processes and molecular functions which are up- and down-regulated. Finally, intersection analysis allowed us to identify the specific features from the oval morphotype which is of particular interest as it is often described to be more resistant to stresses. This study represent the first transcriptome wide characterization of the three morphotypes from *P*. *tricornutum* performed on cultures specifically enriched issued from the same Pt3 strain. This work represents an important step for the understanding of the morphogenesis in *P*. *tricornutum* and highlights the particular features of the oval morphotype.

## Introduction

Diatoms are a major component of phytoplankton communities. They belong to a group of unicellular heterokont microalgae expected to include at least 200,000 species^1^. Their genomes contain unique combinations of nutrient assimilation and metabolic pathways which have been attributed to their origin^2^. Indeed, diatoms derived from a serial secondary endosymbiotic event in which a green algae and subsequently a red one were engulfed by a heterotrophic eukaryote^3–5^. Among the diatom species, *Thalassiosira pseudonana* (*T*. *pseudonana*) and *Phaeodactylum tricornutum* (*P*. *tricornutum*; Pt) have been chosen based on the number of research groups working on them and genome size considerations^6–8^. The comparative analysis of *T*. *pseudonana* and *P*. *tricornutum* genomes highlights the presence of 10% of diatom specific genes which have no homologs in other eukaryotes^6,7,9^. In addition, it has been demonstrated that *P*. *tricornutum* and *T*. *pseudonana* possess 6.4% and 2% of transposable elements, respectively^6,7,10^ as well as microRNAs^11,12^. Moreover, a recent re-analysis of the *P*. *tricornutum* genome revealed that among 251 different patterns identified across the entire genome, many genes have limited evolutionary conservation with 47% having originated within the recent vertical history of the stramenopile lineage and a total of 26% of genes (3170 genes) being specific to *P*. *tricornutum*^8^.

*P*. *tricornutum* is probably the widest studied diatom. Its genome has been sequenced and a large scale transcriptomic analysis has been recently published^7–9^. It is a cosmopolitan diatom not widely distributed in nature, but is encountered principally in coastal unstable environments such as estuaries or rock pools^13,14^. Moreover, *P*. *tricornutum* is able to proliferate in fluctuating environments including various salinity and temperature conditions^13^. These observations highlight the great adaptation capability of this diatom. As previously hypothesized, the adaptation of *P*. *tricornutum* to various environments is probably due to its pleomorphism^13,15–17^. Indeed, *P*. *tricornutum* is an atypical diatom since it can display three morphotypes: fusiform, oval and triradiate^18,19^. As compared to other diatoms, *P*. *tricornutum* presents a high plasticity due to the atypical nature of the cell wall which is poorly silicified^13,15,20^. The fusiform and triradiate morphotypes have previously been described to be poorly silicified whereas the oval morphotype is sensitive to the presence of silicic acid as the oval cells contain organized silicified frustules^20^. In addition, all diatoms reproduce by vegetative division, thus leading to a reduction of the average size of the population^21,22^. Indeed, during the vegetative division, one daughter cell (the cell that inherits the hypotheca of the parent cell) is smaller than the mother cell^22^. When the size of the cell is less than the critical threshold, sexual reproduction (i.e. auxosporulation) trippered. The resulting auxospores will expand and produce cells to restore the cell size of the population. These cells will then reproduce through vegetative division^22^. This predictable change in cell size is called the MacDonald-Pfitzer rule^23–25^. As compared to other pennate diatoms, *P*. *tricornutum* represents an exception to the MacDonald-Pfitzer rule as no cell size reduction has been observed in *P*. *tricornutum* so far. Therefore, auxosporulation seems less crucial for the long term survival of the *P*. *tricornutum* population^21,26,27^. It is possible that this difference in the reproduction process is correlated with the slightly silicified cell wall of *P*. *tricornutum*. Little information is currently available regarding the molecular and cellular biology of *P*. *tricornutum* and especially the physiological significance of its morphogenesis as well as its mechanism of regulation.

Studies have already suggested that the morphogenesis in *P*. *tricornutum* is not dependent on its genotype but is induced by environmental factors^13^. For example, it has been observed that the fusiform morphotype which is the most widespread one and the triradiate are favored in unstressed planktonic environment^13^. It was also noticed that the triradiate morphotype preferentially develops with alkaline condition^28,29^. In contrast, the oval morphotype seems to increase in unfavorable conditions such as hyposaline conditions, low temperature, low light^30^, unshaken liquid culture^16^ or solid substrate^15^. Comparative transcriptomic analysis based on EST showed that the oval morphotype presents up-regulated genes encoding proteins involved in hyposalinity and/or cold stress responses, with putative roles in signaling, membrane remodeling, cryoprotection, osmoregulation, and protein degradation^13^. Furthermore, this form demonstrates an ability to survive with limited nutrient availability which suggests that the oval morphotype could be a resistance form to stresses. More generally, it has been proposed that salinity changes could act as a trigger for changes in morphology^30^.

In this paper, we made use of the power that RNA sequencing (RNA-seq) is offering to identify the whole transcriptome from the fusiform, oval and triradiate morphotypes generated from the same *P*. *tricornutum* Pt3 strain, thus ignoring differences due to the use of different strains and genotypes. The objective of the study was to gain insight into the diatom morphogenesis by identifying genes that are differentially expressed and bringing out specific features which are representatives of each morphotype. A particular attention has been paid to the oval morphotype as it is recognized to be highly resistant to stresses^13,30^.

## Results

### Experimental design and mapping

**Figure 1:**
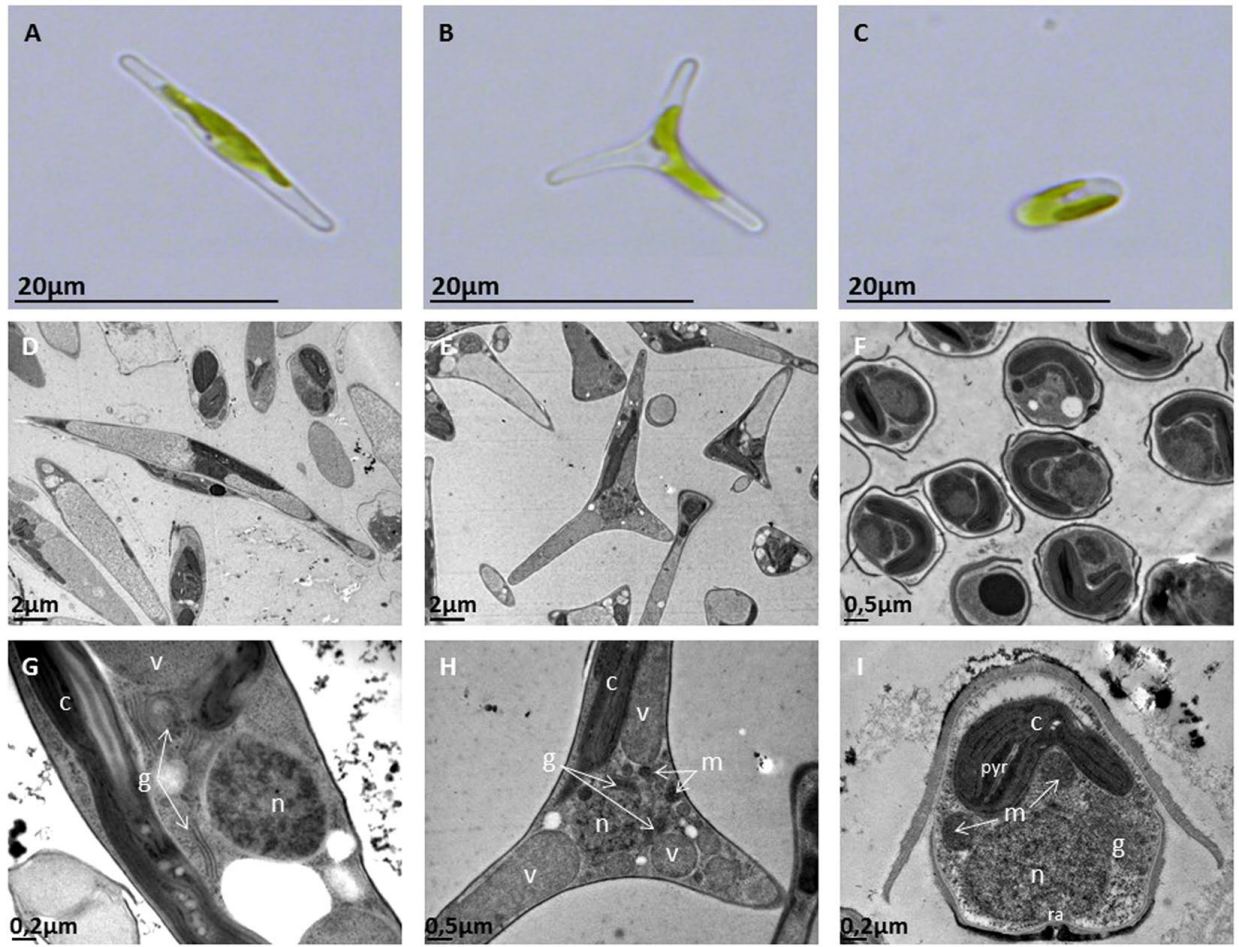
Micrographs of *P*. *tricornutum*’s morphotypes. (**A–C**) Micrographs obtained by light microscopy of *P*. *tricornutum* cells alive. (**A**) fusiform morphotype; (**B**) triradiate morphotype and (**C**) Oval morphotype. (**D–F**) Transmission electron microscopy (TEM) micrographs of the three morphotypes. Overview of the cells which were embedded in LRW resin with 0.5% uranyl acetate in a methanol/Reynold’s lead citrate solution. (**D**) fusiform morphotype; E: triradiate morphotype and (**F**) Oval morphotype. (**G–I**) Enlarge views of the TEM micrographs showing general cellular distribution of organelles in the fusiform cells (**G**), in the triradiate one (**H**) and in the oval cell type (**I**). n: nucleus; g: Golgi apparatus; v: vacuole; m: mitochondria; pyr: pyrenoid; c: chloroplast; ra: raphe.

The Pt3 strain (CCAP 1052/1B; CCMP 2558) used in this study initially derived as a subclonal culture of Pt2 in Plymouth^13^. Originally, this strain contained mostly oval morphotype cells and was able to grow in freshwater media^13^. In this work, in order to evaluate transcriptome changes in *P*. *tricornutum*’s morphotypes, cultures of *P*. *tricornutum* Pt3 strain enriched in one specific and dominant morphotype, either fusiform (91%), triradiate (81%) or oval (94.5%), were generated and followed over a 12-day continuous culture without any change on the morphotype repartition (Figs 1A–F; and S1). The diatom cells were grown as described in the experimental section and follow similar growth curves (Fig. S2). The culture conditions used in this study (100% sea water for the fusiform and triradiate morphotypes and 10% sea water for the oval one) allow us to maintain the dominant specificity of each culture batch (Fig. S1) over a long period of time as well as the diatom cell integrity and organization for each morphotype as illustrated by the TEM micrographs (Fig. 1G–I) and according to the literature^31,32^. Indeed, even if all culture media contained the same amount of silica, the oval morphotype is the only one been extensively silicified and presented one valve with a raphe (Fig. 1)^15,19,30^. The other forms, either fusiform or triradiate have little to no silica (Fig. 1) and no raphe was observed as described previously^13,15–17,31,32^. The ultrastructure of the three morphotypes from *P*. *tricornutum* is similar. Each cell shows a central nucleus. Nearly, we observed from one to four Golgi apparatus. Only one chloroplast is present in the cell, with a central pyrenoid. Chloroplast and mitochondria are closely juxtaposed inside the diatom cell, and mitochondria are tubular.

**Figure 2:**
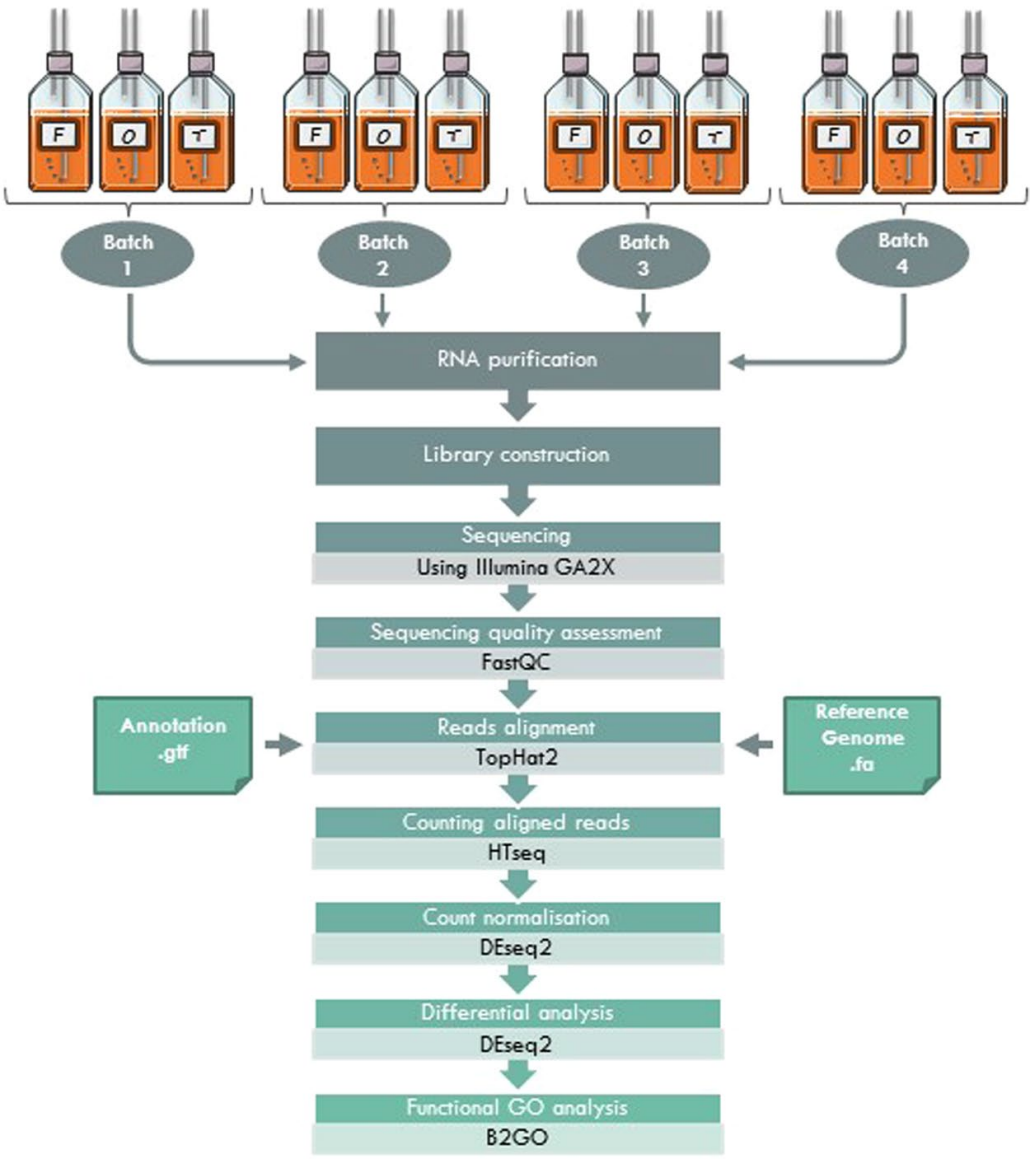
Summary of the experimental design and analytical pipeline used in this study. Briefly, 4 biological replicates of cultures enriched for each specific morphotype were performed in parallel. The RNA were independently purified and used as a matrix to build libraries for RNA sequencing. Quality of the raw data generated have been checked with FastQC. Then, the short reads were aligned on the annotated genome with TopHat2. Aligned reads have been counted with HTseq DESeq2 have been used to normalize counts and to perform the differential analysis. Finally, GO enrichment have been done with B2GO for functional analysis.

**Figure 3:**
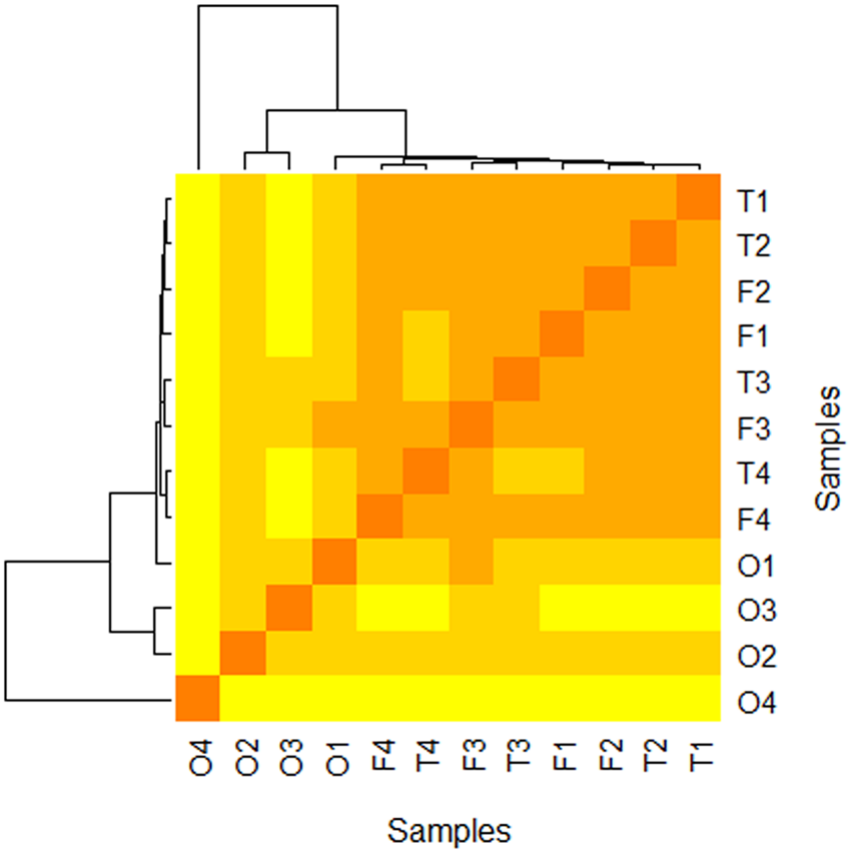
Heatmap illustrating the correlation between the 4 biological replicates for each culture specifically enriched in either the fusiform (F), triradiate (T) or oval (O) morphotype. The heatmap has been built using the distance matrix computation (Additional file 6).

**Figure 4:**
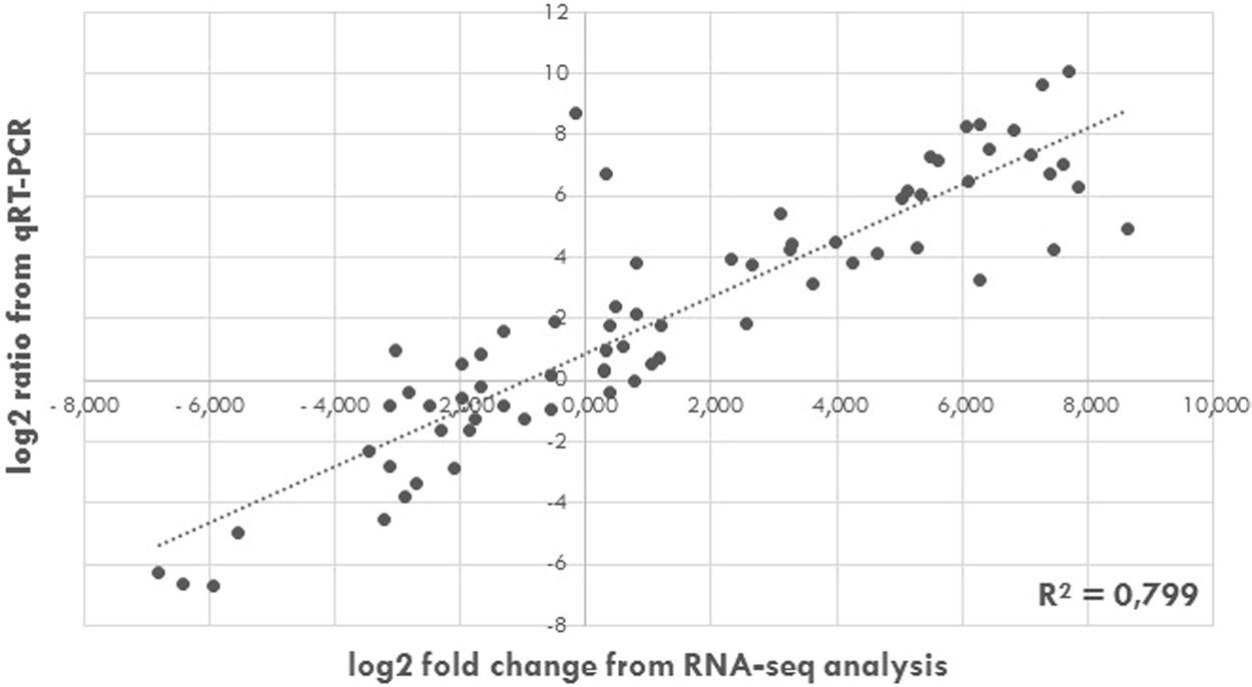
Validation of the RNA-Seq data using qRT-PCR analysis. Correlation between the log2 fold change extracted from the RNA-Seq data and the log2 ratio obtained by qRT-PCR analysis for 22 genes randomly selected from the differentially expressed genes (DEG) lists.

In order to study differential gene expression between *P*. *tricornutum*’s morphotypes and assign specificities for each morphotype, gene transcription profile was investigated using high-throughput RNA-Sequencing (RNA-Seq) following the experimental design which is summarized in Fig. 2. Briefly, 4 biological replicates of each morphotype were cultured for the RNA extraction. No obvious differences were observed in the growth curves at the sampling time of 8 days (Fig. S2). Therefore, the cells were harvested in the middle of the exponential growth phase at day 8 and then further treated for RNA purification and cDNA library preparation in order to perform RNA-Seq analysis. After the sequencing, 38 million quality reads were generated in average per sample. Following the quality assessment, RNA-Seq data were then analyzed using the pipeline described in Fig. 2. The high quality reads were mapped on the Phatr3 annotation version^8^ of the 27.56 Mbp genome of *P*. *tricornutum*’s P.t.1.8.6 strain^7^. Results indicated that around 83% of the RNA-Seq reads were aligned with in average 5% of the reads corresponding to intron/exon junctions for the fusiform morphotype. Similar results were obtained for each morphotype (Figs S3 and S4). Raw reads were counted using HTSeq-count^33^. The read counts were therewith successfully normalized (Fig. S5). As illustrated in Fig. 3 and Fig. S6, biological replicates of the same conditions correlated together by using a distance matrix computation, thus demonstrated the robustness of our experimental design. As a consequence, the biological replicates were averaged for each specific morphotype (n = 4) for the rest of the study. Finally, lists of differentially expressed genes (DEG) were generated using the DESeq2 tool^34^. Only the significantly DEG at an adjusted p-value below 0.01 were conserved. Validation of the RNA-Seq results was performed by qRT-PCR experiments analysing the expression of 22 genes selected randomly from the DEG lists (Table S1). The expression of these genes was normalized to 2 validated reference genes. When compiling the results and plotting the log2 ratio of the qRT-PCR in relation to the log2 fold change from the RNA-Seq, a correlation value R^2^ = 0.799 was obtained, thus attesting of the high quality of our transcriptomic data (Fig. 4).

### Gene differential expression analysis

**Figure 5:**
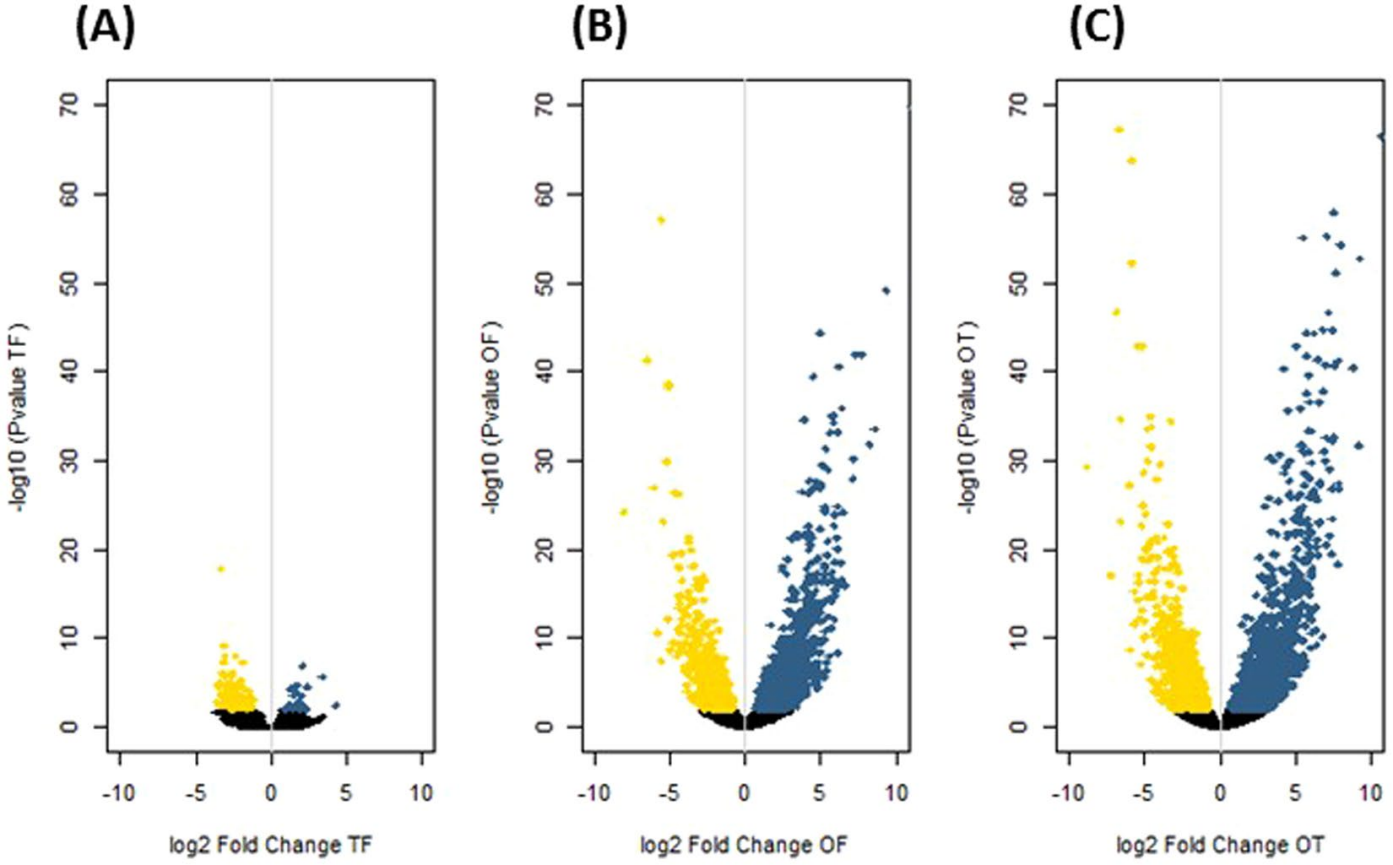
Vulcano Plots of pairwise comparisons of (**A**) T versus F (TF); (**B**) O versus F (OF); (**C**) O versus T (OT). The yellow dots represent down-regulated genes. In contrast, cyan dots represent up-regulated genes. Dark dots represent the non-significantly DE genes.

**Figure 6:**
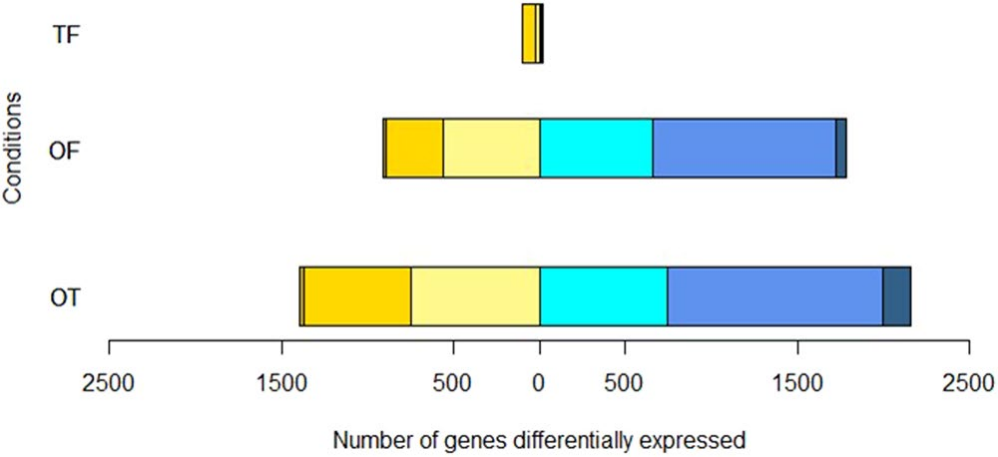
Graph representing the distribution of the significantly differentially expressed genes (DEG) in relation to their log2 fold change values. Blue squares represent up-regulated genes with a log2 fold change value comprised between 0 to 2 for the light blue, 2 to 5 for the median blue and superior to 5 for the dark blue. Yellow squares represent the down-regulated genes with different color intensities which are corresponding to the value of log2 fold change, log2 fold change value comprised between 0 to 2 for the light yellow, 2 to 5 for the median yellow and superior to 5 for the dark yellow.

Pairwise comparisons of the morphotype’s gene expression data were performed by considering that the fusiform morphotype (F) was the reference because it is the most common morphotype found in culture^13,35^. The gene expression profile from the triradiate morphotype (T) was compared first to the one of the fusiform (T *versus* F, noted TF) as these two morphotypes have been previously considered to be the two basic morphotypes in agitated liquid media^28^. Among 12,320 genes described in *P*. *tricornutum*’s genome including 12,178 coding sequences and 142 non-coding, only 120 genes were significantly differentially expressed when comparing TF representing less than 1% of the transcriptome (Fig. 5A and Table S2). As a consequence of this result, considering the small difference observed between T and F, the oval morphotype (O) was thereafter compared to F (OF) and T (OT) respectively. The pairwise comparison of OF highlighted that 2,692 genes were significantly differentially expressed between the two morphotypes, thus representing almost 22% of the transcriptome (Table S2). For the OT comparison, 3,553 genes were described to be significantly differentially expressed representing up to 29% (Table S2). Such results suggest that wide reprogramming of genes is occurring in the oval morphotype as compared to the others. Vulcano plot for TF shown that 100 genes are down-regulated in the triradiate morphotype with the majority inside the interval of the log2 fold change values comprised between 2 to 5 (Figs 5 and 6). When focusing on the OF comparison, 906 genes are down-regulated and 1,786 genes are up-regulated in the oval morphotype. As far as the up-regulated genes are concerned in the OF DEG list, 662 genes are differentially expressed at less than a value of 2 for the log2 fold change and 1,059 being differentially expressed at a log2 fold change comprised between 2 to 5. Only 65 genes are up-regulated at a value greater than 5. The down-regulated genes of OF are mostly greatly than the log2 fold change value 5, with only 11 genes being differentially expressed at a log2 fold change lower than 5, 332 genes comprised between 2 to 5 and 563 genes having a log2 fold change comprised between 0 to 2. The OT comparison reveals 3,553 DEG with 1,396 being down-regulated and 2,157 being up-regulated. When looking at the log2 fold change value, 744 of the up-regulated genes are in the 0 to 2 range for their log2 fold change value, 1,255 genes are between 2 to 5 and 158 genes display a value higher than 5. The down-regulated genes possess 746 genes with a value comprised between 0 to 2 and 624 genes comprised in the interval 2 to 5. Only 26 genes are down-regulated at a log2 fold change value greater than 5 for the OT comparison (Figs 5 and 6).

### Functional profiling analysis

**Figure 7:**
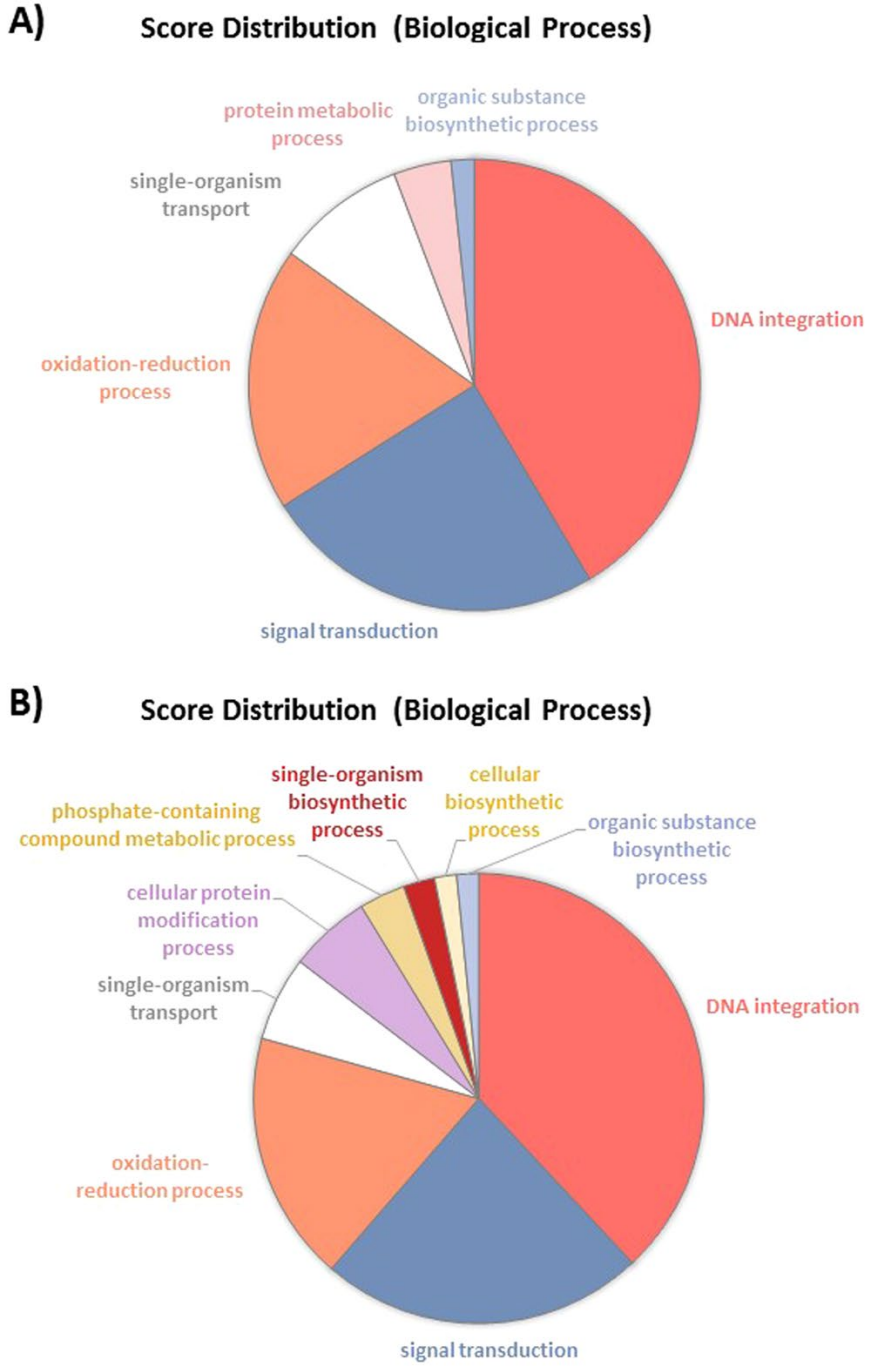
Pie charts representing the biological processes which are alliterated in the TF pairwise comparison. (**A**) Overall biological processes associated to up- and down-regulated genes and (**B**) biological processes which are specifically associated to down-regulated genes.

In order to characterize the biological processes (BP) and molecular functions in which DEG are involved, a gene ontology (GO) term analysis was performed using Blast2GO^36^ (see experimental procedure for details). From this analysis, above 65 to 70% of the transcripts from RNA-Seq data have been annotated with GO terms. The pie chart of the TF comparison shown that some biological processes are regulated (Fig. 7). Among them, DNA integration represents about 41% of the DEG genes which are almost all down-regulated. Signal transduction BP is enriched to 25%, being also down-regulated. The oxidation-reduction process is also appearing as 19% of the overall down-regulated genes. Genes dedicated to single-organism transport accounted for 9% of the DEG and are also mainly down-regulated. Finally, protein metabolic process (4%) and organic substance biosynthetic process are completing the overall and down-regulated pie charts, representing 4 and 2% respectively (Fig. 7). Similar results were obtained when considering the score distribution at the molecular function level (Fig. S7). Indeed, for example, above 40% of the down-regulated genes are corresponding to nucleic acid binding activity, 17% coincide with signal transducer activity, 16% represent protein binding and 10% of the down-regulated genes are represented by alternative oxidase activity. Altogether, these results reinforce the ones achieved for the biological process.

**Figure 8:**
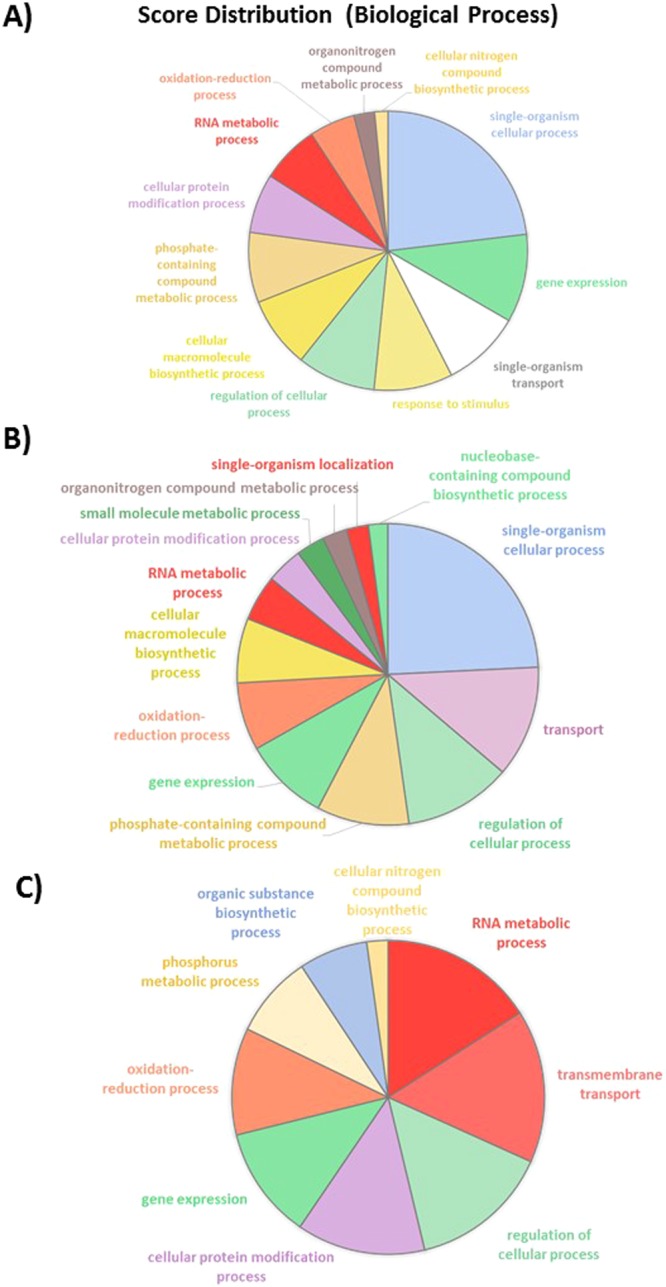
Pie charts representing the biological processes which are alliterated in the OF pairwise comparison. (**A**) Overall biological processes associated to up- and down-regulated genes and (**B**) biological processes which are specifically associated to up-regulated genes, (**C**) biological processes associated to down-regulated genes.

As already mentioned, 2,692 genes are differentially expressed in the OF pairwise comparison with 906 down-regulated genes and 1,786 up-regulated genes. Among these genes, 23% are corresponding to single-organism cellular process with the majority being up-regulated, 10% belong to the gene expression, 9% to the single-organism transport, 9% to the response to stimulus and to regulation of cellular process, 8% to the cellular macromolecule biosynthetic process and to phosphate containing compound metabolic process (Fig. 8). In addition, 7% represent respectively cellular protein modification process and RNA metabolic process. Oxidation-reduction process, organonitrogen compound metabolic process, cellular nitrogen compound biosynthetic process represent less than 5% for each category. In contrast, RNA metabolic process seems to be more down-regulated. Genes involved in the regulation of cellular process are equally distributed between the up- and down-regulated genes. Similar repartition is observed for genes involved in the oxidation-reduction process. In contrast, genes implicated in the cellular macromolecules biosynthetic process and organonitrogen compound metabolic process are all up-regulated whereas cellular nitrogen compound biosynthetic process and cellular protein modification process are down-regulated (Fig. 8). The analysis at the molecular function level shows that transferase activity, hydrolase activity, nucleic acid binding, protein binding, metal ion binding and oxidoreductase activity are equally classified in the up- and down-regulated sub-categories (Fig. S8). In contrast, genes dedicated to the purine binding (purine ribonucleotide binding, purine ribonucleoside triphosphate binding and purine ribonucleoside binding) are all down-regulated (Fig. S8).

**Figure 9:**
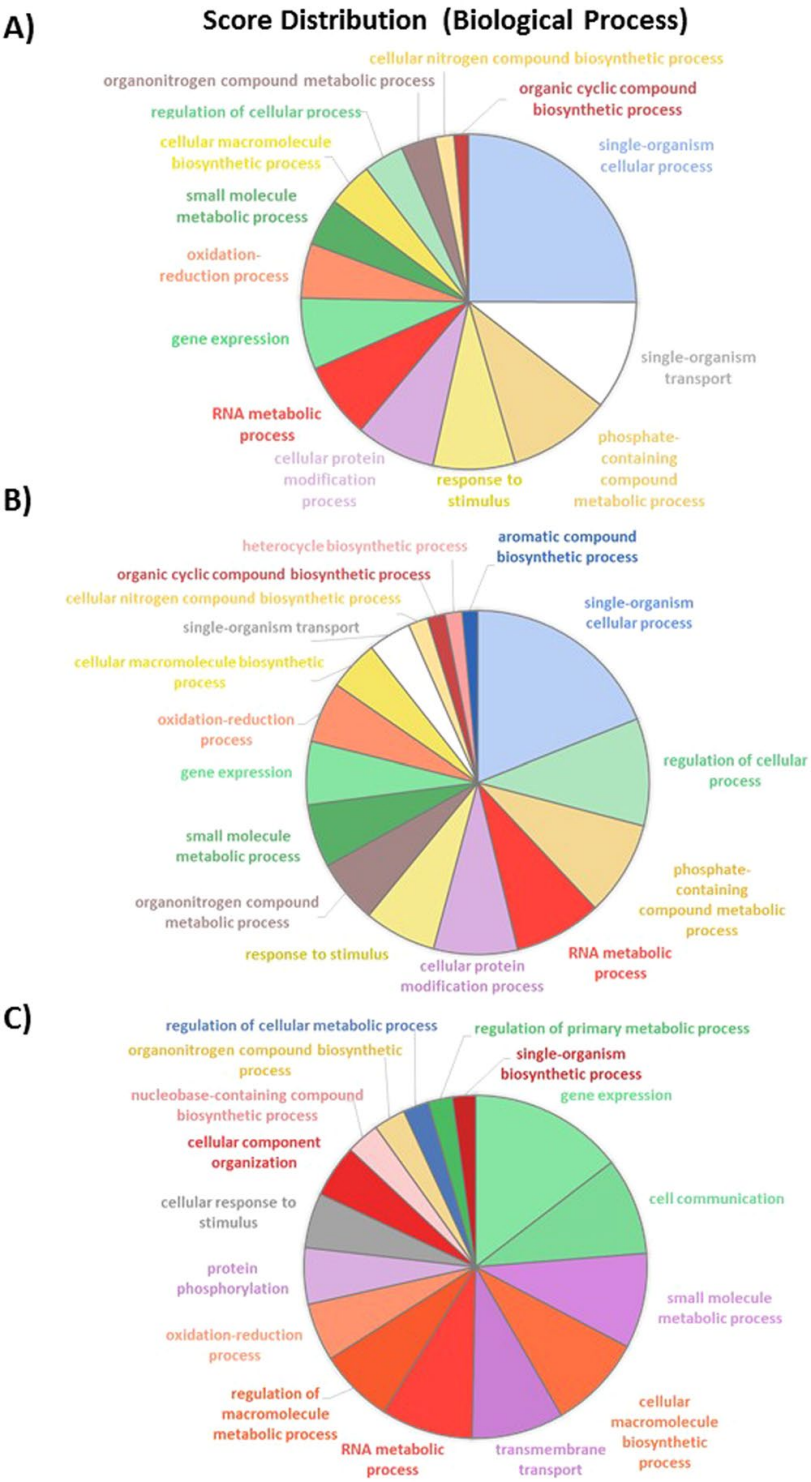
Pie charts representing the biological processes which are alliterated in the OT pairwise comparison. (**A**) Overall biological processes associated to up- and down-regulated genes and (**B**) biological processes which are specifically associated to up-regulated genes, (**C**) biological processes associated to down-regulated genes.

Among the 29% of the transcriptome thus representing 3,553 genes which are significantly differentially expressed in the OT pairwise comparison, 25% correlated with single-organism cellular process. Such BP seems to be mainly up-regulated in the OT condition (Fig. S8). Some of the DEG are implicated in single-organism transport (11%) being mostly up-regulated as it is for the phosphate-containing compound metabolic process (10%), response to stimulus (8%), cellular protein modification process (8%) and cellular nitrogen compound biosynthetic process (2%) (Fig. 9). By opposition, oxidation-reduction process is equally distributed between the up- and down-regulated genes and the gene expression BP is mostly down-regulated (15%) as it is for cellular macromolecule biosynthetic process (9%). When looking at the molecular function analysis, it appears that the hydrolase activity representing 18% of the overall molecular function is mostly down-regulated (27%). The nucleic acid binding and the oxidoreductase activity are equally represented in both the up- and down-regulated gene lists (Fig. S9). The transferase activity is only present in the up-regulated genes category. An opposite situation appears for the ATP binding, kinase activity, transporter activity which are only down-regulated. The metal ion binding function is present in both cases with a majority of genes being up-regulated (Fig. S9).

### Intersection analysis reveals specific pathways which are up-regulated in the oval morphotype

**Figure 10:**
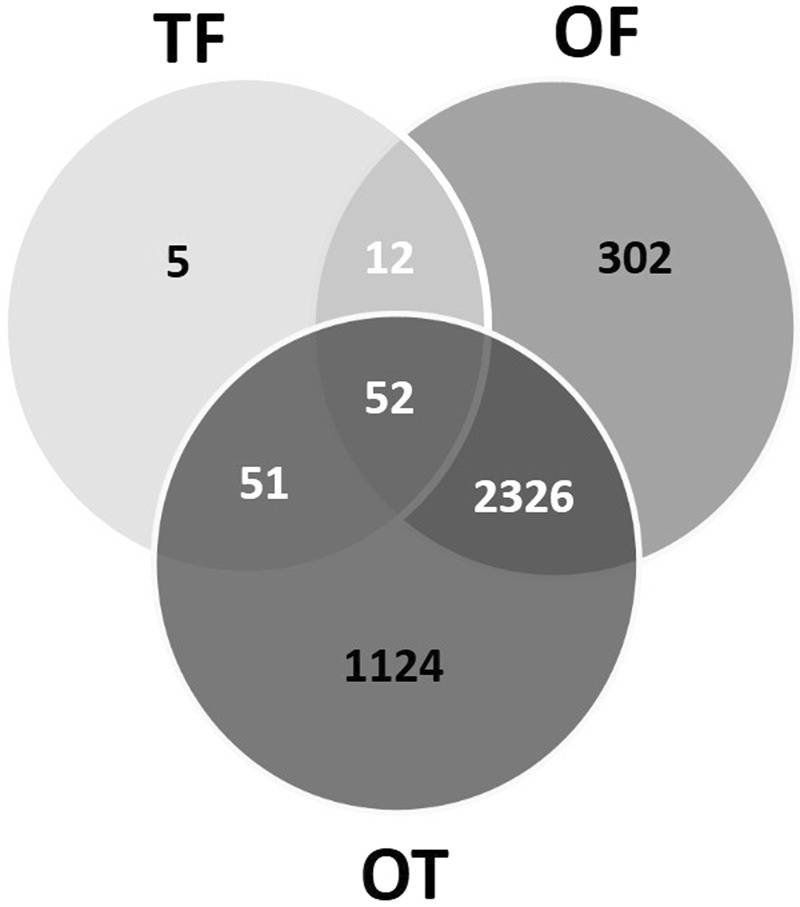
Venn diagram showing the distribution of the DE genes between the three pairwise comparisons.

**Figure 11:**
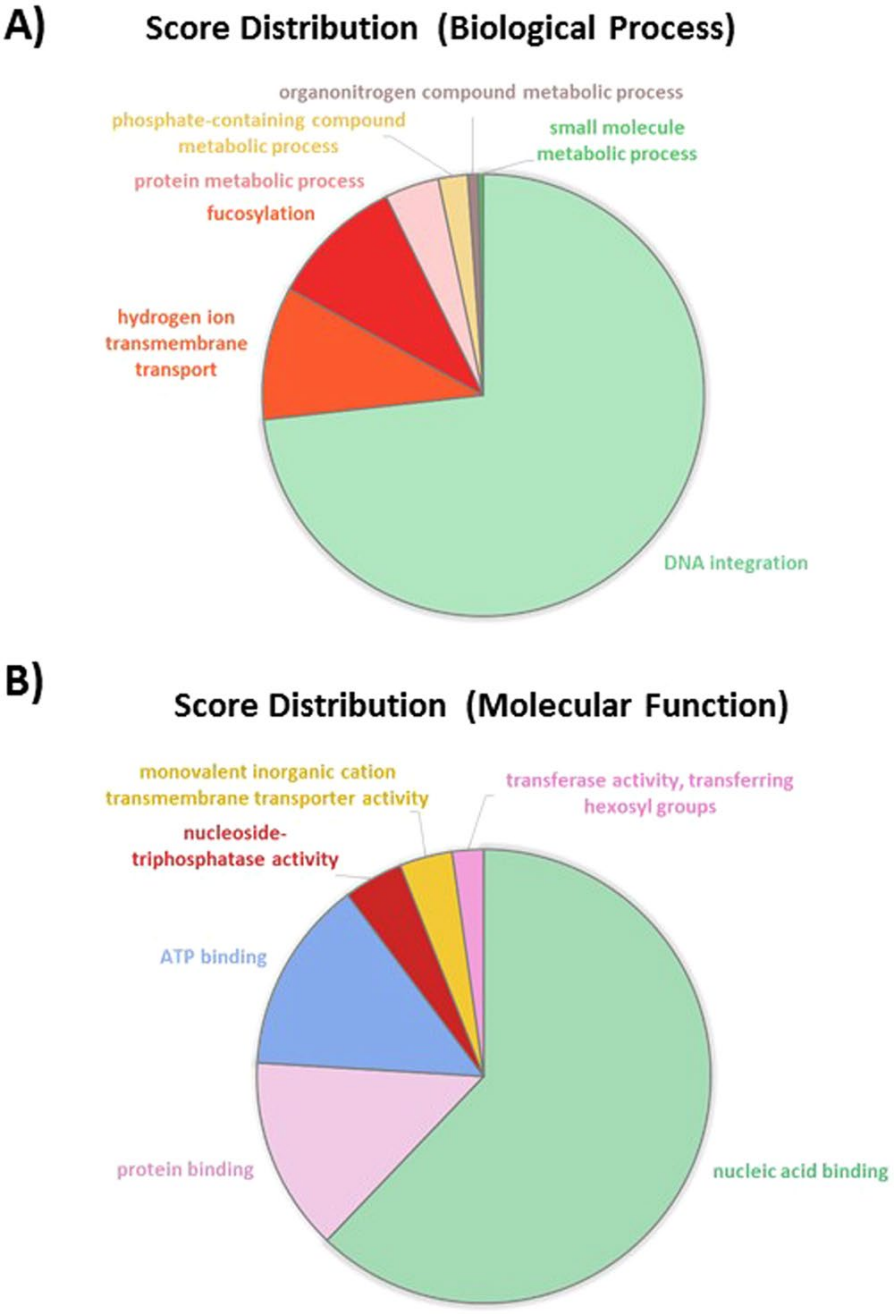
Pie charts representing the biological processes and molecular functions which are alliterated in the 52 common genes between TF, OF and OT. (**A**) Overall biological processes associated to up- and down-regulated genes; (**B**) overall of the molecular functions associated to up- and down-regulated genes.

Intersection analysis was performed in order to identify specific genes which can be attributed to one morphotype. The resulting Venn diagram is presented in Fig. 10. It demonstrated that 52 genes are common to all pairwise comparisons. These genes are differentially expressed in a common manner for the three morphotypes. However, when looking precisely at the log2 Fold Change, this one is reflecting a specific gene expression level for each morphotype (Table S3). Among the list, 17 genes were not annotated with any GO term. 18 genes out of the 52 are encoding proteins related to nucleic acid binding functions with a very high percentage (almost 70%) being DNA polymerases (Pol proteins). This fact is well illustrated in the score distribution pie charts which came out from the analysis of the BP and molecular function distribution through Blast2GO analysis. Indeed, DNA integration and nucleic acid binding occupy the biggest part of the pie charts, respectively 73% and 62% (Fig. 11).

Coming back to the Venn diagram (Fig. 10), it is noticeable that only 5 genes came out to be specific of the TF pairwise comparison. Among the GO terms associated, transferases activity, carbohydrate and nucleic acid binding proteins can be found. The TF and OF comparisons overlap and present 12 common genes which have either no attributed GO term (5 genes) or relative to signal transduction and DNA integration process (7 genes). Similarly, 51 genes show up to overlap between TF and OT (Table S3), thus being specific of the triradiate morphotype. 18 out of the 51 genes are encoding putative proteins which currently do not have any associated GO terms. Among the rest, signal transduction and DNA integration represent almost 45% of both the BP and molecular function distributions. Oxido-reduction process, transmembrane transport and protein phosphorylation are also enriched (Table S3).

**Figure 12:**
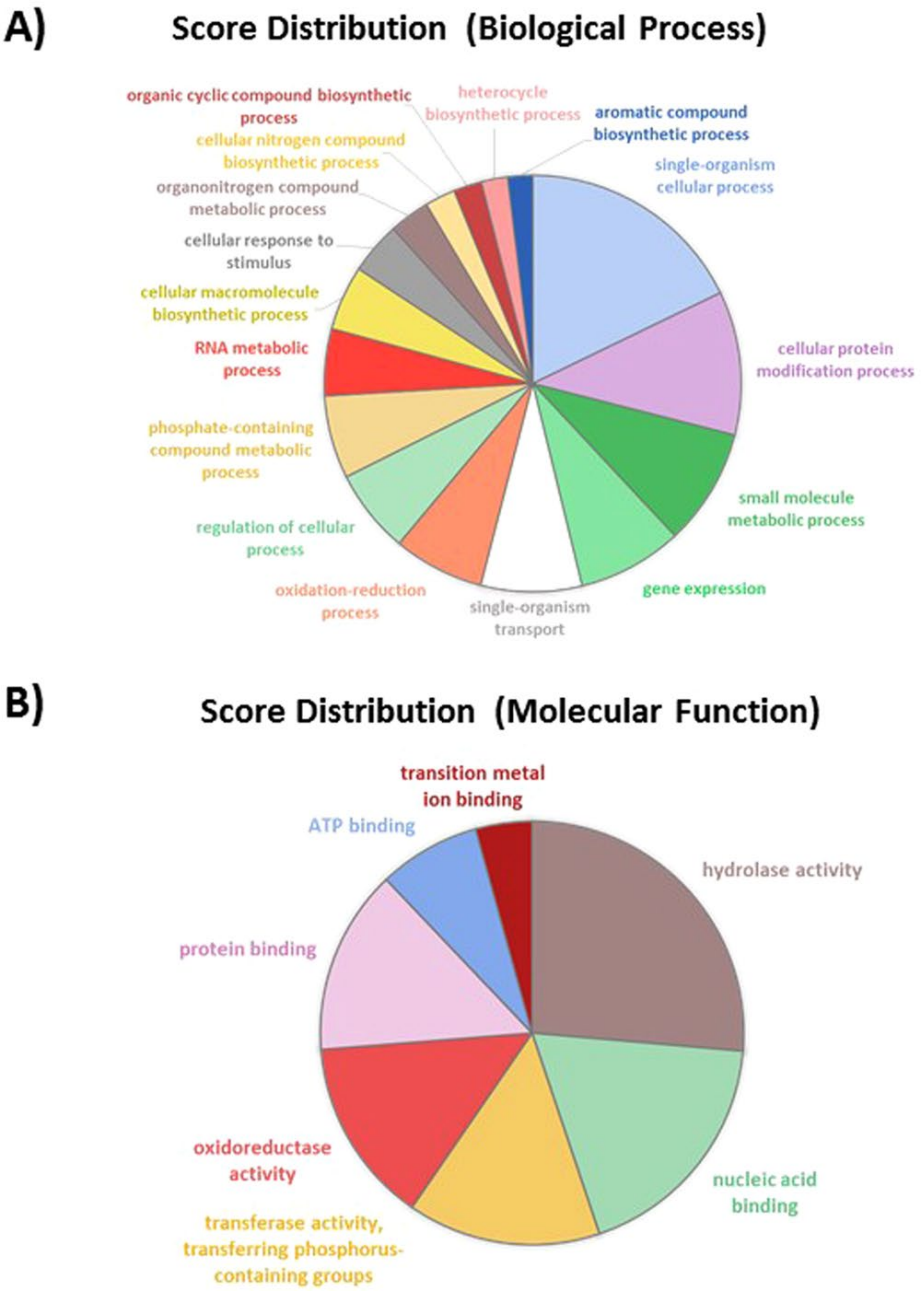
Pie charts representing the biological processes and molecular functions which are alliterated in the 2,336 genes specific to the oval morphotype. (**A**) Overall biological processes associated to up- and down-regulated genes; (**B**) overall of the molecular functions associated to up- and down-regulated genes.

In order to identify genes which are specifically down- or up-regulated and pathways affected in the oval morphotype, a particular attention has been paid to the analysis of the 2,326 DEG which are at the intersection of the OF and OT pairwise comparisons (Table S3). Among this DEG list, 68% of the genes were up-regulated. For this dataset, a GO and a pathway analysis were run in parallel independently. Results of the GO analysis and enrichment for both the biological processes and molecular functions are summarized in Fig. 12. The pie charts show that the single-organism cellular process (18%), the cellular protein modification process (11%), the small molecule metabolic process (9%), the gene expression (8%) and single organism transport (8%) represent major biological processes which are modified within the oval morphotype from *P*. *tricornutum*. The analysis at the molecular function level highlighted changes in the hydrolase activity (26%), nucleic acid binding (18%), transferase activity (15%), oxidoreductase activity (14%), protein (14%) and ATP binding (8%) as well as transition metal ion binding (4%).

In parallel, genes encoding known enzymes in the *P*. *tricornutum*’s genome were mapped in KEGG (Kyoto Encyclopedia of Genes and Genomes) pathways using both B2GO and Pathview^37–40^. Among the 2,326 genes from the DEG list, 1,862 are possessing a UniProt annotation and only 1,483 sequences are having an attributed EC numbers. The pathway analysis supports a putative activation of key primary metabolism pathways such as the glycolysis, the pentose phosphate pathway and the biosynthesis of nucleotides and triglycerides in the oval morphotype of *P*. *tricornutum* (Table S3). Indeed, the depolymerisation of chrysolaminarin, the diatom storage β(1,3)-glucan, by a set of glucanases into free glucose and then its subsequent phosphorylation by hexokinase into glucose 6-phosphate allows to supply the glycolysis and the pentose phosphate pathway in carbon sources. Then, overexpression of the glyceraldehyde-3-phosphate dehydrogenase (Phatr3_J 23598; EC:1.2.1.59), that catalyzes the synthesis of glycerate 1,3 bis-phosphate in the glycolysis, as well as the overexpression of sub-units of the pyruvate dehydrogenase allows the conversion of glucose 6-phosphate into acetyl CoA for the carbon supplementation of the citrate cycle. In addition, nucleotide biosynthesis is likely triggered in the oval morphotype of *P*. *tricornutum*. For instance, overexpressions of the glucose-6-phosphate dehydrogenase (Phatr3_EG02298; EC:1.1.1.49), 6-phosphogluconolactonase (Phatr3_J8691; EC:3.1.1.31; EC:3.1.1.1) and 6-phosphogluconate dehydrogenase (Phatr3_J45141; EC:1.1.1.44; EC:1.1.1.31 and Phatr3_EG02298; EC:1.1.1.44; EC:1.1.1.49) of the cytosolic pentose phosphate pathway reflect the activation of the ribose phosphate biosynthesis that is required for the synthesis of both nucleotides and deoxynucleotides (Table S3). Genes encoding key enzymes involved in the biosynthesis of purines and pyrimidines are also overexpressed in the oval morphotype.

Analysis of the expression of genes encoding enzymes of the lipid pathways mainly revealed a down-regulation in the oval morphotype of genes encoding enzymes specific of the fatty acid degradation together with the overexpression of the diatom phosphatidic acid phosphatase as well as monoacyl and diacylglycerol O-acyltransferases. Thus, this suggests that triglyceride biosynthesis is activated in this morphotype. Among other gene families exhibiting differential expression between *P*. *tricornutum* morphotypes, it is worth noting that a hexokinase (Phatr3_J48173; EC:2.7.1.4; EC:2.7.1.1) is overexpressed in the oval diatom. This enzyme is able to supply the cytosol in mannose 6-phophate that is required for the biosynthesis of GDP-Man nucleotide sugar. This may support an active biosynthesis of the glucuronomannans in this morphotype. Indeed, glucuronomannans have been previously described to be the main polysaccharide component of *P*. *tricornutum*’s cell wall^41–43^. In addition, the gene encoding for the glutamine fructose 6 phosphate transaminase (Phatr3_J14994; EC:2.6.1.16) is down-regulated, therefore limiting the synthesis of L-glutamate and D-glucosamine-6-phosphate. Meanwhile, the phosphoribosyldiphosphate 5-amidotransferase (Phatr3_J37436; EC:2.4.2.14) is up-regulated meaning that the L-glutamine is used to synthesize 5-phospho ribosylamine which enters into the purine metabolism. This example illustrates again that the oval morphotype promotes purine synthesis pathway at the expense of the aminosugars metabolism.

**Figure 13:**
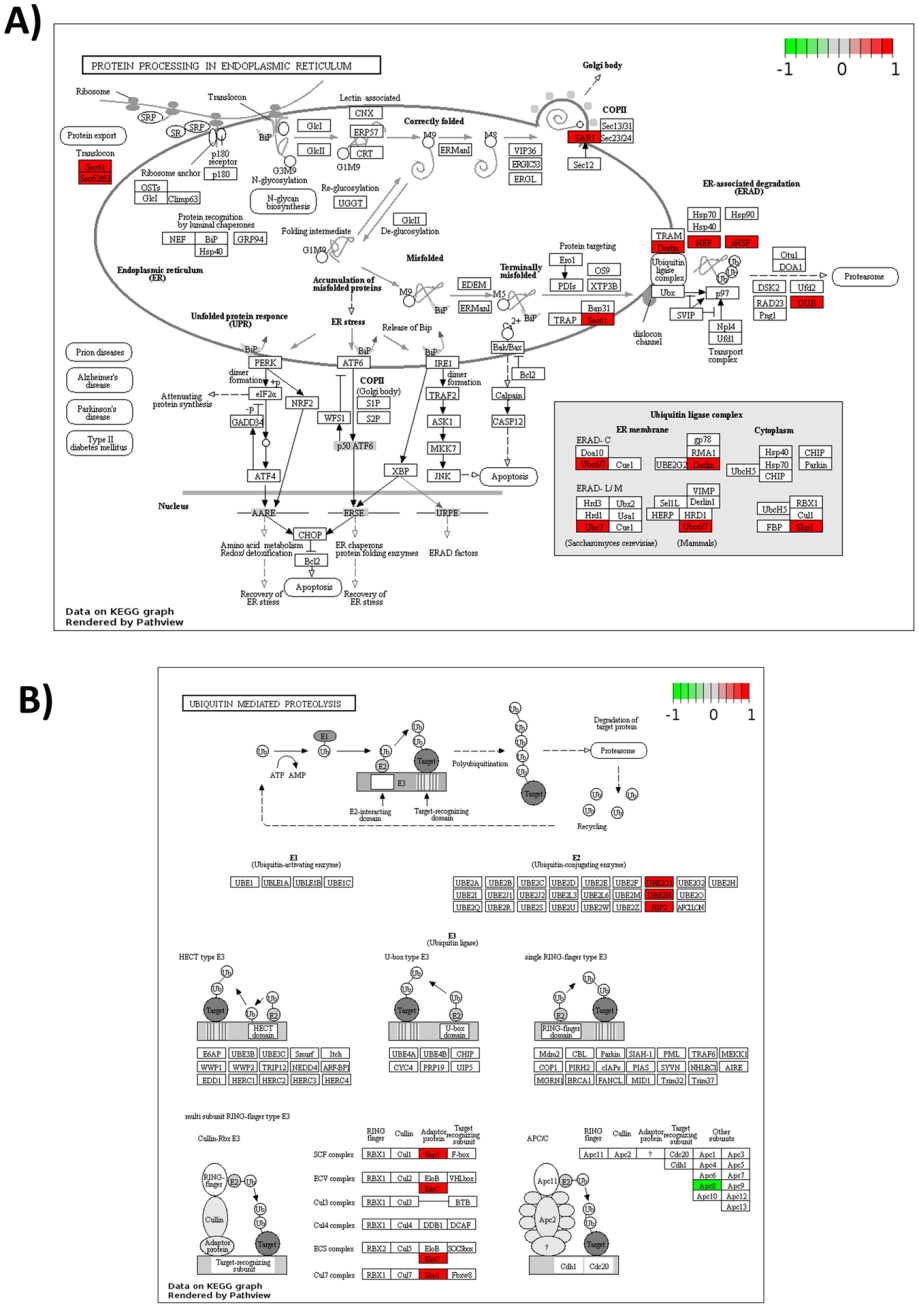
Mapping of the DE genes exclusive to the oval morphotype on KEGG metabolic pathways^38–40^. (**A**) Protein processing in endoplasmic reticulum pathway; (**B**) Ubiquitin mediated proteolysis pathway; (**C**) Proteasome pathway. Copyright permission of KEGG pathway maps were kindly provided by the Kanehisa laboratory.

**Figure 14:**
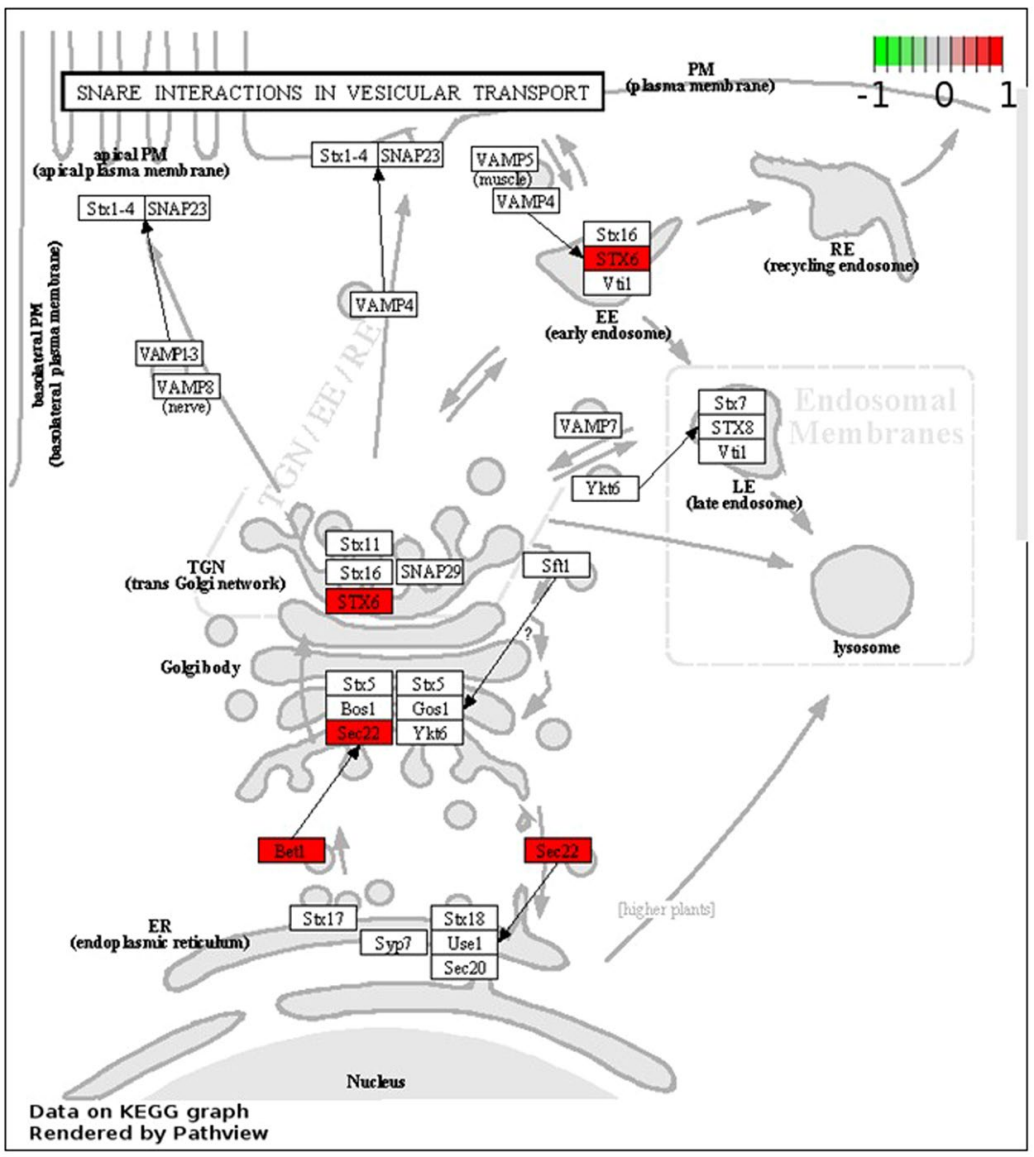
Mapping of the DE genes exclusive to the oval morphotype on the SNARE interactions in vesicular transport pathway issued from the KEGG metabolic pathway^38–40^. Copyright permission of this KEGG pathway map was kindly provided by the Kanehisa laboratory.

The metabolism of some of the amino acids is also modified within the oval morphotype (Table S3). For example, the Serine biosynthesis is up-regulated whereas its transformation into glycine is down-regulated leading overall to an increase of Serine. The global pool of Cysteine is also boosted. Indeed, the Serine is transformed in L-Cysteine through the overexpression of the gene encoding the cysteine synthase A (Phatr3_EG01318; EC:2.5. 1.47) and the formation of the acetyl-L-Serine intermediate. The synthesis of the D-cysteine is intensified through the up-regulation of the gene encoding for an amino acid racemase (Phatr3_J13987; EC 5.1.1.10). Besides the regulation of the Serine and Cysteine metabolism, some of the steps of the Arginine biosynthesis are also down-regulated leading to a decrease of glutamate and citruline which are both precursors of Arginine and Aspartate. The gene encoding for the acetolactate synthase I/II/III large subunit (Phatr3_J37341; EC 2.2.1.6) is also up-regulated. This enzyme transforms pyruvate in S-2-aceto 2 hydroxybutanoate, an intermediate of the L-isoleucine biosynthesis. The tyrosine aminotransferase, the aromatic amino acid transaminase, the aspartate transferase and the histidinol-phosphate aminotransferase (Phatr3_J38891; EC 2.6.1.5; EC 2.6.1.57; EC 2.6.1.1; EC 2.6.1.9) are down-regulated. All together, these steps induce a reduction of the accumulation of L-glutamate, thus favoring the synthesis of Phenylalanine and Tyrosine. In agreement with this and the impact observed on Isoleucine metabolism, the overexpression of genes encoding the phenylalanyl-tRNA synthetase (Phatr3_J33783; EC 6.1.1.20) and the isoleucyl-tRNA synthetase (Phatr3_EG00945; EC 6.1.1.5) can be noticed. In complement, an overexpression of genes encoding the alanyl-tRNA synthetase (Phatr3_J35813; EC 6.1.1.7), the lysyl-tRNA synthetase class I (Phatr3_J46593; EC 6.1.1.6) and the methionyl-tRNA synthetase (Phatr3_J13445; EC 6.1.1.10) can be observed, even if the Lysine and Methionine metabolism are not affected. In constrast, genes encoding for the aspartyl-tRNA synthetase (Phatr3_J42051; EC 6.1.1.12) and the asparaginyl-tRNA synthetase (Phatr3_EG02403; EC 6.1.1.22) appears to be significantly down-regulated. As the aminoacids metabolism and aminoacyl tRNA biosynthesis were perturbed, we decided, then, to focus our analysis on the protein metabolism. The KEGG and Pathview analysis demonstrated that the cellular protein modification process are up-regulated, thus confirming the results of the previous BP and molecular function studies. Indeed, such analyses demonstrated that several actors involved in the protein degradation processing are up-regulated (Fig. 13). Among them, genes involved in protein processing in the endoplasmic reticulum (ER) are up-regulated (Fig. 13A). This is the case for the Sec61/Sec62/Sec63 (Phatr3_J17885; Phatr3_J47099) complex which consists of membrane protein translocators allowing the translocation of a neo-synthesized polypeptide within the ER^44,45^. In parallel, genes like Ubc6/7 (Phatr3_J28433), Derlin (Phatr3_J6076; Phatr3_J28348), skp1 (Phatr3_J17276) encoding for proteins belonging to the Ubiquitin ligase complex^46,47^ and genes encoding for actors of the ER-associated degradation like Derlin, NEF (Phatr3_J47062), sHSF (Phatr3_J35158), DUB (Phatr3_J47158) that detects misfolded proteins in the ER and targets them for destruction^48^ are also up-regulated. In addition, the ubiquitin-mediated proteolysis mechanism is also up-regulated (Fig. 13B) as well as some genes encoding for protein subunits of the proteasome (Fig. 13C). Thus, taken together, those results suggests that the protein degradation metabolism is much more active in the oval morphotype as compared to the others and it can be hypothesized that in *P*. *tricornutum*, like in other eukaryotes, Sec61/Sec62/Sec63 complex might also be responsible of the backward transport of ER proteins in order to submit them to the ubiquitin-proteasome dependent degradation pathway. We noticed that in the KEGG pathway corresponding to the protein processing in the ER, the gene encoding for SAR1 (Phatr3J_54420), a GTPase specifically found in COP II vesicles, was up-regulated (Fig. 13A). Therefore, we decided to focus on the vesicular transport (Fig. 14) and observed that the gene encoding for Bet1 (Phatr3J_13691), a Golgi vesicular membrane trafficking protein was up-regulated. This protein is a Golgi-associated membrane protein that participates in vesicular transport from the ER to the Golgi complex. In human, it has been demonstrated that this protein functions as a SNARE involved in the docking process of ER-derived vesicles with the *cis*-Golgi membrane^49,50^. Additionally, Sec22 (Phatr3J_54066), a gene encoding for a SNARE involved in targeting and fusion of ER-derived transport vesicles with the Golgi complex as well as Golgi-derived retrograde transport vesicles with the ER^51,52^ is up-regulated. This particular SNARE has been recently described to be responsible for the regulation of the ER morphology in *Drosophila*^53^. Therefore, once can hypothesize that the ER morphology of *P*. *tricornutum*’s oval morphotype needs to be maintained to face the overactivity of the cell machinery. In our experiment, the gene encoding for the Syntaxin 6 (STX6; Phatr3J_31559) was also up-regulated (Fig. 14). STX6 is a Q_bc_-SNARE^54^. It displays an important role in protein trafficking between the *trans*-Golgi network (TGN) and the ensodomal system^55–57^. Its up-regulation in the oval morphotype suggests that the *P*. *tricornutum* cells are secreting more materiel for degradation through the endosomal system as compared to the other morphotypes.

As a validation experiment, the gene expression data for the oval morphotype were compared to the ones of T and F combined together. This comparison came out with a similar DEG list as the one containing the 2,336 differentially expressed genes generated from the Venn diagram (data not shown).

## Discussion

This work is aiming at studying and comparing the transcriptomes of the three morphotypes from *P*. *tricornutum*, namely the fusiform, oval and triradiate one^15,18,19,32^. In order to get the most accurate transcriptomic comparisons avoiding any influence of genotype differences, the Pt3 strain^12^ was adapted to generate enriched cultures in each specific morphotype. Such enrichment reached more than 80% for the triradiate morphotype culture and more than 90% for the fusiform and oval cultures. Even if it was not possible to reach 100% for each morphotype, the above mentioned percentage seems to be high as compared to those given in previous articles. Indeed, for example, Maheswari and co-workers in ref.^58^ generated an ESTs database on the three morphotypes of *P*. *tricornutum* issued either from the Pt3 strain for a culture enriched up to 80% in oval; from the Pt8 strain for a culture enriched up to 70% in triradiate and finally Pt1.8.6 for a culture containing more than 95% of the fusiform cells^58^. One year later, De Martino and collaborators reported evidence that environmental changes elicit morphological interconversion in *P*. *tricornutum*^30^. This study was performed using cultures of Pt1.8.6 containing 80% of the fusiform morphotype and cultures of Pt3 containing 60% of oval cells^30^. The temperature used for these cultures was 19 °C similarly to what was used for the Pt3 cultures in the current work. The mentioned previous studies were performed by using different strains as representative culture of one specific morphotype. In contrast, in the present work, we have chosen to generate morphotype enriched culture all issued from the Pt3 strain as it was clearly demonstrated that different accessions of *P*. *tricornutum* were significantly different^13^, thus introducing potential biases within the results. In addition, the enriched cultures for one dominant morphotype generated for this study were stable over several months and years as previously described^13,15,18,19,59^. This was achieved by decreasing the percentage of sea water to 10% for the culture containing high percentage of the oval morphotype. All the other components of the media including silicate remained the same. Indeed, in our hands, it was not possible to maintain for more than 1 week the enriched culture in oval morphotype using 100% sea water media as previously described^30,58^. Therefore, it is important to keep in mind that such differences in culture conditions (100% versus 10% sea water) might induce differences in gene expression in the oval morphotype due to the decrease in salinity. However, the differences in salinity did not affect the integrity of the cellular organization which was confirmed by TEM for the three morphotypes and was consistent with data previously described in the literature^15,31,32^. Our experimental plan (Fig. 2) was designed to combine recommendations for the RNA-Seq data analysis best practices as recently reviewed by Conesa and collaborators^60^. To demonstrate the robustness of the RNA-Seq data obtained in this study, 22 DEG were randomly selected and their respective expression levels were analyzed by qRT-PCR. The correlation between the qRT-PCR results and the RNA-Seq measurement came out with a R^2^ value equal to 0.799, thus supporting the high quality and consistency of the dataset.

Pairwise comparisons of the morphotype’s gene expression data were performed by considering that the fusiform morphotype (F) was the reference because it is the most common morphotype found in culture^13,35^. Therefore, the comparison TF, OF and OT were done respectively. Only small differences representing less than 1% of the transcriptome were observed in the TF comparison as only 120 genes were significantly differentially expressed between these two morphotypes. This is in agreement with the report of ref.^28^ which considered the fusiform and triradiate morphotypes from *P*. *tricornutum* as two basic morphotypes. More differences were observed in the OF and OT pairwise comparisons. Indeed, 2,692 and 3,553 genes were significantly differentially expressed in these conditions. Such results represent 22 and 29% of the transcriptomes respectively. About 83.3% of the genes are down-regulated in the TF condition whereas 67% of the genes are up-regulated in OF and 62% are up-regulated in OT, suggesting that the oval morphotype might be more metabolically active. Amid these genes, 30 to 35% does not have any associated GO term. This could probably be due to the fact that the genome of *P*. *tricornutum* contains specific genes related to diatom metabolism. Indeed, previous reports indicate that at least 10% of the diatom genomes correspond to diatom specific genes^6,7,9^. Even if not complete, the GO annotation obtained in this work seems to be better than previous studies which have reported that only half of the diatom genes can be assigned to a putative function using homology–based methods^61^. Similarly, Rogato and co-workers^62^ suggested recently that half of the diatom genes encodes proteins of unknown functions. Hereafter, more investigations need to be done in order to characterize the DEG which still do not have any associated GO term. Surprisingly, lots of these genes are presenting high value of log2 fold change being in the extreme parts of the vulcano plots (Fig. 5).

The GO annotation and intersection analyses revealed global changes in major categories of genes involved in various pathways when comparing two by two the morphotypes from *P*. *tricornutum*. A particular focus has been done on the analysis of the 2,326 DEG which are present at the intersection of the OF and OT pairwise comparisons, thus being characteristic of the oval morphotype. Within the 2.326 DEG, 68% were found to be up-regulated, thus suggesting that the metabolism should be more active in the oval morphotype which is known to be resistant to stresses^13^. In our hands, the oval morphotype pathway analysis supports an activation of key primary metabolism pathways such as the glycolysis, the pentose phosphate pathway and the biosynthesis of nucleotides and triglycerides similarly to what has been described in the fusiform morphotype when studying the lipid accumulation mechanisms following nitrogen deprivation^63^. Altogether, these results suggested that the triglyceride biosynthesis is activated in the oval morphotype, that the glucuronomannan biosynthesis is more active and that the oval morphotype promotes nucleotide synthesis pathway. Such increase of the triacylglyceride biosynthesis pathway has been previously reported when Pt1 strain from *P*. *tricornutum* was cultured in media depleted in nitrate and exogenous phosphate^64^. As far as the glucuronomannan biosynthesis is concerned, one can hypothesize that the oval morphotype which is resistant to stresses need to protect itself with a bigger layer of cell wall. Indeed, glucuronomannan has previously been described to represent the major polysaccharide component of the cell wall from *P*. *tricornutum*^31^. *P*. *tricornutum* cells, like all other eukaryotic cells, contain a conventional endomembrane system composed of the nuclear envelope, the ER, the Golgi apparatus and the *trans* Golgi network which are connected to each other by vesicular shuttles^65^. When looking at the specific activity of the endomembrane system, we noticed that the protein processing in the ER was overexpressed jointly with some actors from the Ubiquitin ligase complex as well as genes involved in the ER-associated degradation process which encode for proteins involved in the detection of misfolded proteins and their targeting for destruction. In the same guideline, the ubiquitin-mediated proteolysis mechanism, the proteasome and vesicular transport are also up-regulated. Such results demonstrate that, in the oval morphotype, more proteins are secreted and sent for degradation through the endosomal system as compared to the other morphotypes.

In the future, the RNA-Seq data generated in this work will be used to identify and characterize transcripts whose GO gave no results as well as novel transcripts, alternative splicing analysis and polymorphism in *P*. *tricornutum*. This could complete a recent study which demonstrated that extensive alternative splicing involved in regulation of gene expression in response to nutrient starvation is occurring in *P*. *tricornutum*^8^. In addition, such analysis will help unravel rare and novel transcripts and detect eventual layer of regulation never studied so far in *P*. *tricornutum*. Additionally, it should allow *de novo* transcript reconstruction which could help refining the quality of the genome annotation in complement to what has been recently done with large-scale RNA-seq transcriptomes and EST datasets^8^. It would also be interesting to analyze and compare the small RNAs transcriptome and the DNA methylation status of the three morphotypes, and evaluate whether the oval morphotype is presenting some differences when compared to the other morphotypes. Indeed, Rogato and co-workers demonstrated that small RNAs, including ncRNAs, could play important functional roles when *P*. *tricornutum* fusiform morphotype was grown at 18 °C under different conditions of light and iron starvation^62^. These results suggest that small ncRNAs may be fundamental for the biology of *P*. *tricornutum* especially when responses to stresses are concerned^62^. Therefore, it can be tempting to think that the oval morphotype may have a different profile of small ncRNA compared to the other morphotypes in *P*. *tricornutum*. This hypothesis is coherent with the fact that in our findings some actors from the spliceosome (Sm; SF3b; Prp38; DIB1; SPF27; PPIL1; data not shown) are up-regulated in the oval morphotype. In addition, it would be of interest to study and compare the gene expression of the three morphotypes depending of the different growth phases (latence, exponential and stationary) to evaluate whether significant differences occur during the growth phases of the three morphotypes as compared to the mid-exponential growth phase which was chosen in this paper. It would also be interesting to extend this study by comparing two different strains from a different geographical origin using a molecular barcoding. Finally, proteomic study comparing the three morphotypes from *P*. *tricornutum* in different conditions would confirm and complement the present transcriptomic finding and oval specific features at the protein level. Previous results^16^ suggested from comparative SDS-PAGE experiments that the fusiform and oval protein patterns are slightly different. Based on the DEG lists that were obtained in the current RNA-Seq analysis, we can expect much more differences at the protein expression level when comparing the oval proteome to the one from the fusiform and triradiate respectively. The quality and power of such proteomic approach should be increased if the proteome used as a reference is based and enriched in reconstructed transcripts. Therefore, proteogenomics which intends to identify novel peptides and proteins (not present in the reference protein sequence databases) from mass spectrometry–based proteomic by using a customized protein sequence databases generated from genomic and transcriptomic information^66^ could now be considered in *P*. *tricornutum*. In complement, proteomic data from *P*. *tricornutum* could be used to refine gene models based on protein-level evidence of gene expression.

## Materiel and Methods

### Culture and growth conditions

*Phaeodactylum tricornutum* strain Pt3 (CCAP 1052/1B; CCMP 2558) was used for this study^13^. Cultures enriched in fusiform, oval or triradiate morphotypes were adapted in the laboratory. Diatom cells were grown at 19 °C in 1 L bioreactors on a 16 h/8 h light/night cycle. The intensity of the light used was 68 µmol.m^−2^.s^−1^. The nutritive medium used was composed of natural seawater 100% for the fusiform and triradiate morphotypes and 10% for the oval morphotype. It was sterilized by filtration through a 0.22 µm filter, autoclaved, and then enriched in Conway containing 80 mg.L^−1^ of sodium metasilicate (Na_2_SiO_3_) as described in ref.^67^. The diatom cells were cultured under ambient air at a regulated flow rate of 0.83 L.min^−1^. CO_2_ from the air was the only available source of carbon for the diatoms. Bioreactors were inoculated with a final microalgae concentration of 1 × 10^6^ cells.mL^−1^. A cell count was performed daily on a Nageotte chamber. This cell count was done in triplicate for each culture during 15 days to follow the cell growth (Figs S1 and S2). According to this protocol, cultures containing a dominant morphotype were done in parallel. Four biological replicates for each morphotype’s culture were performed independently (Fig. 2).

### RNA extraction

In order to isolate total RNA, cells were collected at day 8, in the middle of the exponential growth phase (Fig. S2). For each culture, 100 mL (8.10^8^ cells) were recovered and centrifugated during 3 min at 10,000 g at 19 °C. Cell pellets were immediately resuspended in 1 mL of TRIzol® Reagent (Ambion by Life Technologies) and the suspension transferred in a 2 mL tube of lysing matrix E (MP Biomedicals) and immediately flash frozen in liquid nitrogen. The samples were then stored at −80 °C until RNA extraction. For RNA extraction, cells were lysed by 4 runs of 30 sec at 6.5 m.s^−1^ with the FastPrep®−24 high-speed benchtop homogenizer (MP Biomedicals). Samples were stored on ice between each run to avoid RNA degradation. Cell lysates were then incubated at room temperature during 5 min prior to a 5 min centrifugation at 14,000 g. Then, RNA was isolated using the TRIzol® reagent by using the standard protocol given by the supplier (Ambion®, Life Technlogies) followed by a purification on Nucleospin RNA II column (Macherey-Nagel) including an on-column DNAse I treatment and finally eluted with 60 µL of RNAse-free water and stored at −80 °C until library preparation. For each preparation, the RNA concentration was determined in duplicate using a Nanodrop spectrophotometer (Thermo Scientific). In addition, the RNA quality was controlled using an Agilent 2100 Bioanalyser (Agilent Technologies).

### Library preparation and RNA-sequencing

Libraries were prepared from 4 µg of total RNA using the Illumina Truseq® Stranded mRNA Sample Preparation kit according to the manufacturer’s protocol (Illumina). Briefly, mRNAs were purified and fragmented before first strand synthesis, second strand synthesis was performed and adaptors were ligated to the products before PCR amplification of the samples as previously recommended to minimize bias^68^. Indexed libraries were normalized and pooled two by two for random multiplexing. Libraries were subjected to a 75 bp paired-end sequencing on an Illumina GAIIx, according to the manufacturer’s protocol. Two samples were pooled on a flowcell lane. Raw image files were processed using the Illumina Real Time Analysis (RTA 1.9) software (Illumina). Demultiplexing was performed using CASAVA 1.8.2 (Illumina). On average, 38 millions of reads were generated per sample corresponding to 2.9 Gb per sample. 94% of bases were read with a Qscore above 30. Raw data are available at the European Nucleotide Archive, accession number PRJEB26173 (http://www.ebi.ac.uk/ena/data/view/PRJEB26173).

### Bioinformatic analysis

#### Quality assessment

Quality assessment of the short reads was realized with FastQC v0.10.1 (S. Andrews, FastQC a quality-control tool for high-throughput sequence data, http://www.bioinformatics.babraham.ac.uk/projects/fastqc/2010) (Fig. S10). Over represented sequences were found to correspond to adapters. In a first attempt, various trimming assays were tested using either the FASTX-Toolkit (FASTQ/A short-reads pre-processing tools, http://hannonlab.cshl.edu/fastx_toolkit/) or a home procedure with perl scripting. Such treatment of the data had no impact on the results of the mapping. Therefore, the trimming step was not necessary and we decided to keep all the reads without trimming for further steps.

#### Read alignement

Raw reads were aligned to the *P*. *tricornutum* reference genome sequence (.FA), and the reference annotations (.GTF), both obtained from http://protists.ensembl.org/Phaeodactylum_tricornutum (release 25, dec 2014). The total genome sequence (JGI-Phatr2, Assembly ASM15095v2 Feb 2010; INSDC Assembly GCA_000150955.2) contains 27,568,093 bp including 33 finished nuclear chromosomes (26,138,756 bp), the chloroplast chromosome (117,369 bp) and 55 unmapped sequences (Phatr2_bd, 1,311,968 bp). All were included in this study for comprehensive analysis. Although transcripts from the chloroplast lacking polyA tail are supposed to be automatically removed due to the purification method of the mRNA by polyA selection, we nonetheless decided to include the chloroplastic genome. EnsemblProtists annotations (.GTF file) used for alignment include 12,178 protein coding genes (12,392 gene transcripts), 177 non-coding genes and 37 pseudogenes. RNA short reads were aligned to the reference genome using the splice junction mapper TopHat 2 v2.0.6 (ccb.jhu.edu/software/tophat/)^69^ with the option “-library-type fr-firststrand”, and Bowtie v2.0.2^70,71^ with default parameters with the exception of Max-multihits = 1.

#### Read count and normalization

For gene expression quantification, the resulting alignment files for each condition, and the reference annotation (.GTF file) guiding the transcript count values, were provided to HTSeq-Count v0.5.4p1^33^ used with the intersection-nonempty mode. For each gene defined in the Phatr2 database, counts were normalized with DESeq2 v1.14.0^34^; https://bioconductor.org/packages/release/bioc/html/DESeq2.html).

#### Differential analysis

In order to carry out differential analysis, DESeq2 v1.14.0^34^; https://bioconductor.org/packages/release/bioc/html/DESeq2.html) was used with the option “fittype = local” to perform pairwise comparisons between samples and find significantly differentially expressed genes. The differential expression analysis in DESeq2 uses a generalized linear model where gene counts are modeled using a negative binomial distribution. The adjusted p-value was obtained by the Benjamini-Hochberg multiple testing adjustement procedure which is known to counteract the problem of multiple comparisons and to control the False Discovery Rate^72^.

#### Functional analysis and enrichment

Prior to perform such analysis, EPr sequences corresponding to ncRNA (tRNA, rRNA, snoRNA) have been removed manually from the dataset. For each significantly differentially expressed genes (P adjusted value < 0.01), GO annotation and enrichment were performed using Blast2GO v3.3.5^36^; https://www.blast2go.com) with default parameters Intersection analysis was performed with Venny v2.1 (http://bioinfogp.cnb.csic.es/tools/venny/). Pie charts were done with Multi-Level option in Blast2GO considering the biological process and molecular function. In order to perform mapping on metabolic pathways, significantly DEG were mapped with the help of Pathview^37^; http://pathview.uncc.edu), and the mapping tool of Blast2GO powered with KEGG database.

### qRT-PCR Validation

The High capacity cDNA reverse transcription kit from Applied Biosystems was used to carry out the cDNA synthesis with total RNA samples from the four biological replicates of the three different morphotypes following the manufacturer’s instructions. One µg of total RNA purified as described above was used per reaction in a final volume of 20 µL, using random primers as cDNA priming strategy. The cDNAs were then stored at −20 °C until use.Twenty-two genes of interest (GOI) were randomly selected from the RNA-seq data analysis, picked from the 3 DEG lists resulting from the pairwise comparisons of *P*. *tricornutum*’s morphotypes. Primer pairs specific to each GOI were designed by using the Primer-BLAST tool at NCBI (http://www.ncbi.nlm.nih.gov/tools/primer-blast/). Validation steps of each primer pair in order to control its specificity and evaluate its amplification efficiency were performed. The quantitative real-time PCR was carried out with the QuantStudio^TM^ 12 K Flex Real-Time PCR System (Applied Biosystems) and the pipetting steps were done with the Bravo Automated Liquid Handling Platform (Agilent Technologies). The reaction mix were prepared in a 96-well reaction plate using the Fast SYBR Green Master Mix (Applied Biosystems) in a total volume of 13 µL. The final primer concentration in the reaction mix is 100 nM each and the cDNA volume added is 3 µL. The following amplification protocol was used: 20 s at 95 °C then 40 cycles of 3 s at 95 °C, 20 s at 60 °C followed by the melt-curve analysis: 15 s at 95 °C, 1 min at 60 °C and 15 s at 95 °C. The qRT-PCR data were analyzed accordingly by using a combination of the methods described by ref.^73^ and ref.^74^. In the first one, a derivation of the 2^−∆∆Ct^ is used to estimate the expression level of a target gene taking into account the amplification efficiency of the primer pair used specifically for this gene. According to the second method, this gene expression value is then normalized by using the geometric mean of the 2 best reference genes, selected among a set of candidates by a Normfinder analysis^75^, as normalization factor (Table S4).

### TEM microscopy for P. tricornutum morphotype characterization

#### High pressure freezing (HPF) and freeze substitution (FS)

HPF was performed with the freezer HPF-EM PACT I Leica-microsystems^76^. Prior to freezing, *P*. *tricornutum* suspension-cultured cells were treated 2 hours with 100 mM mannitol as cryoprotectant diluted in culture medium at room temperature. Pre-treated *P*. *tricornutum* cells were then transferred into the cavity of a copper ring used for cryofracture, which is 100 µm in depth and 1.2 mm in diameter. Excess medium was lightly absorbed by filter paper. Using a horizontal loading station, the specimen carriers were tightened securely to the pod of specimen holder. After fixation on the loading device, specimen were frozen according to a maximum cooling rate of 10,000 °C*sec^−1^, incoming pressure of 7.5 bars and working pressure of 4.8 bars. Rings containing frozen samples were stored in liquid nitrogen until the freeze substitution procedure was initiated.

#### Freeze substitution

After high-pressure freezing, samples were transferred to a freeze substitution automate (AFS, Leica, http://www.leica-microsystems.com) pre-cooled to −140 °C. Freeze substitution conditions followed a modified procedure from ref.^76^. Samples were substituted in anhydrous acetone with 0.5% uranyl acetate at −90 °C for 96 h. The temperature was gradually raised to −60 °C using a gradient of +2 °C.h^−1^ and stabilized during 12 h, then gradually raised to −30 °C by using the same gradient during 12 h and gradually raised again to −15 °C for 8 h. Finally, samples were rinsed twice with anhydrous ethanol.

Infiltration was then processed at −15 °C in a solution containing ethanol: London Resin White (LRW) in a ratio 2:1 overday; 1:1 overnight and 1:2 overday and then 100% of a pure resin solution overnight. This solution was changed twice and incubated for 2 times 24 h. The LRW was finally polymerized into the AFS apparatus at −15 °C under ultra violet light during 48 h.

Ultrathin sections (80 nm; ultracut UCT, Leica) of *P*. *tricornutum* cells were collected onto carbon-formvar-coated nickel grids. A classical staining using uranyl acetate and lead citrate was done before sections were observed in a Philips, FEI Tecnai 12 Biotwin transmission electron microscope operating at 80 kV, with ES500W Erlangshen CCD camera (Gatan).

## Electronic supplementary material

Supplemental Figures

Supplementary Dataset 1

Supplementary Dataset 2

Supplementary Dataset 3

Supplementary Dataset 4

## Data Availability

The analyzed data are presented within the different tables and figures of this paper. Raw data are available at the European Nucleotide Archive, accession number PRJEB26173.
