# Supplementary material for: Comparative in depth RNA sequencing of P. tricornutum’s morphotypes reveals specific features of the oval morphotype
Source: Sci Rep. 2018 Sep 25;8:14340. doi: 10.1038/s41598-018-32519-7 (PMC6156597; doi:10.1038/s41598-018-32519-7)
Supplement: Supplementary file 1 — Supplemental Figures [file 41598_2018_32519_MOESM1_ESM.docx]

**Comparative in depth RNA sequencing of *P. tricornutum*’s morphotypes reveals specific features of the oval morphotype.**

Clément Ovide^1^***,** Marie-Christine Kiefer-Meyer^1^*****, Caroline Bérard^2^, Nicolas Vergne^3^, Thierry Lecroq^2^, Carole Plasson^1^, Carole Burel^1^, Sophie Bernard^1,4^, Azeddine Driouich^1,4^ Patrice Lerouge^1^, Isabelle Tournier^5^, Hélène Dauchel^2^***^♯^** and Muriel Bardor^1, 6^***^♯^**

**** These authors equally contributed to the work.***


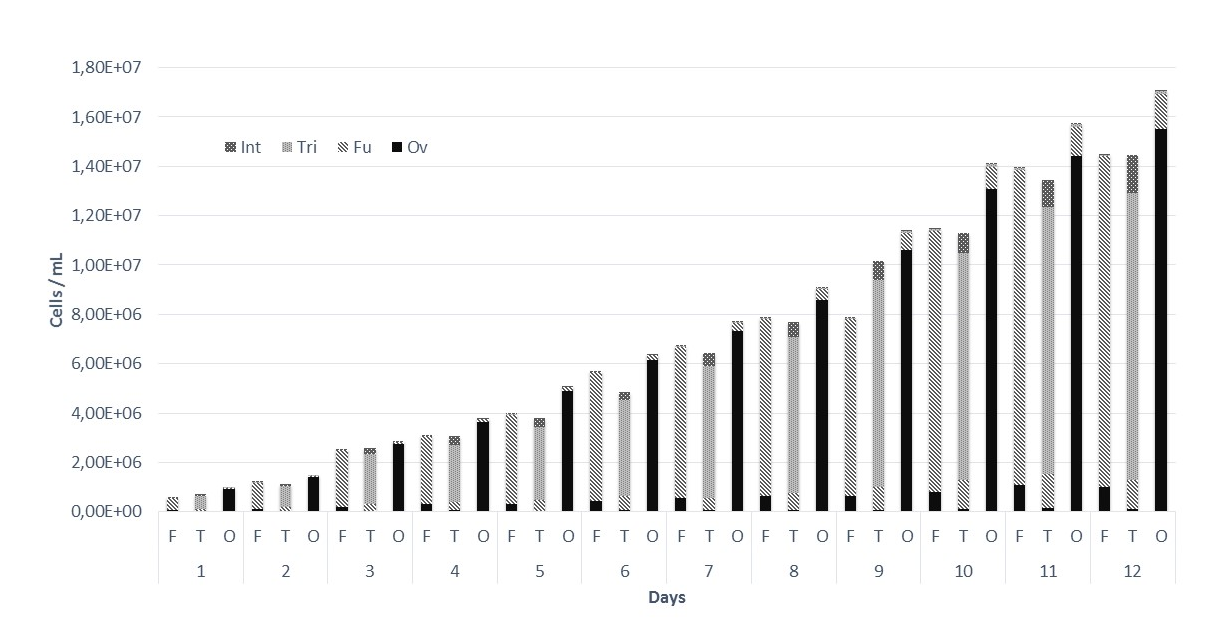


**Figure S1:** Cells and morphotypes count in the cultures of *P. tricornutum* over a 12-day period.


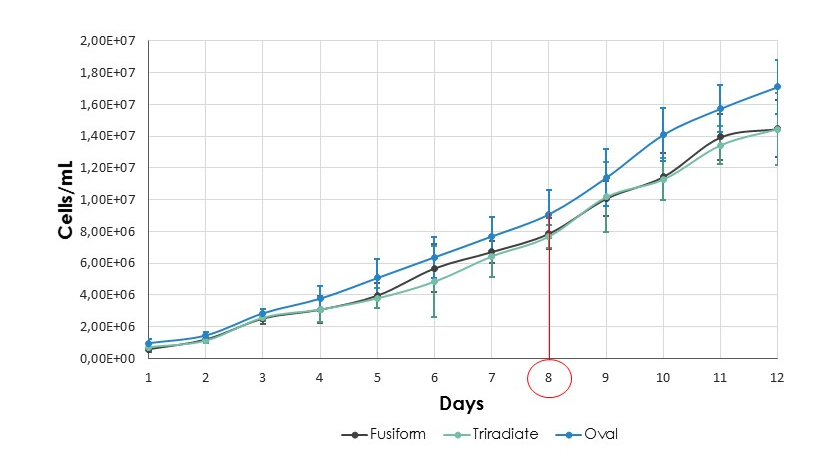


**Figure S2:** Growth curves of the fusiform, oval and triradiate enriched cultures over a 15-day period. Cells have been harvested at day 8 in the middle of the exponential growth phase for RNA purification.


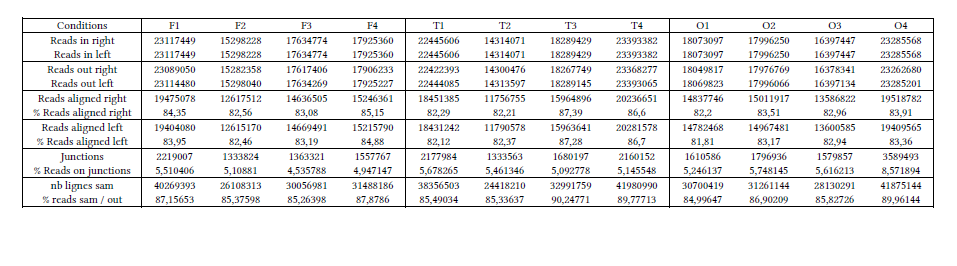


**Figure S3.** Metric of mapping from TopHat for the 4 biological replicates of the cultures enriched in the fusiform, triradiate and oval morphotype, respectively.


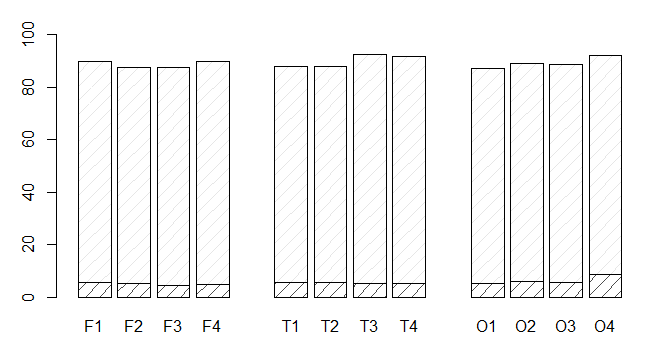


**Figure S4:** percentages of read alignment with TopHat for the 4 biological replicates of the cultures enriched in the fusiform, triradiate and oval morphotype, respectively. Proportion of reads aligned to intron-exon junction are also indicated at the bottom of the bars.


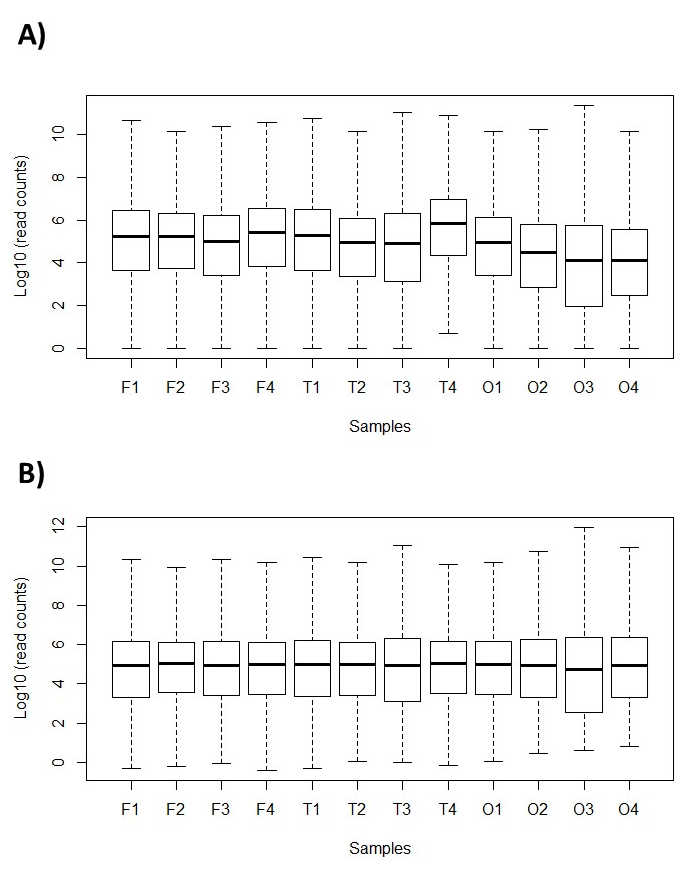


**Figure S5.** Correlations between biological replicates and normalization performance with DEseq2. A) Correlation before DEseq2 normalization and B) Correlation after DEseq2 normalization.

**Supplemental Table S1:** Comparative table highlighting the RNA-Seq log2 fold change and the qPCR log2 ratio for the 22 genes randomly selected among the DEG list obtained from the TF; OF; OT pairwise comparison of the RNA-Seq analysis.

**Supplemental Table S2:** List of the genes significantly differentially expressed (adjusted p-value below 0.01) in the OF, OT and TF pairwise comparisons.

**Supplemental Figure S6.** Results of the matrix distance computation performed after the normalization with DEseq2.


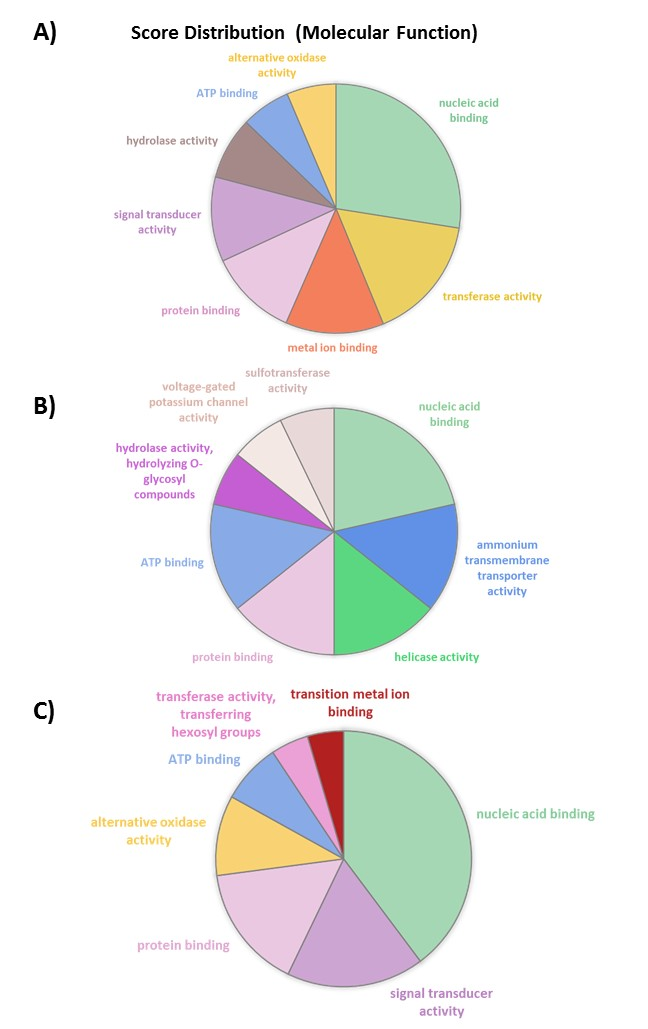


**Supplemental Figure S7:** Pie charts representing the molecular functions which are alliterated in the TF pairwise comparison. (A) Overall of the molecular functions associated to up- and down-regulated genes; (B) molecular functions associated to up-regulated genes and (C) molecular functions associated to down-regulated genes.


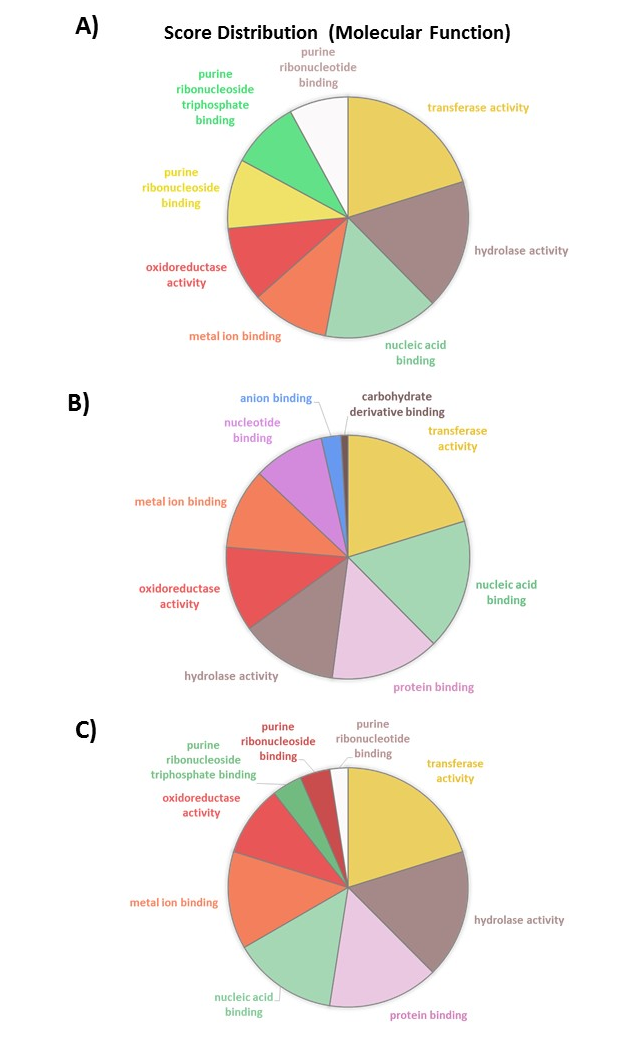


**Supplemental Figure S8:** Pie charts representing the molecular functions which are alliterated in the OF pairwise comparison. (A) Overall of the molecular functions associated to up- and down-regulated genes; (B) molecular functions associated to up-regulated genes and (C) molecular functions associated to down-regulated genes.


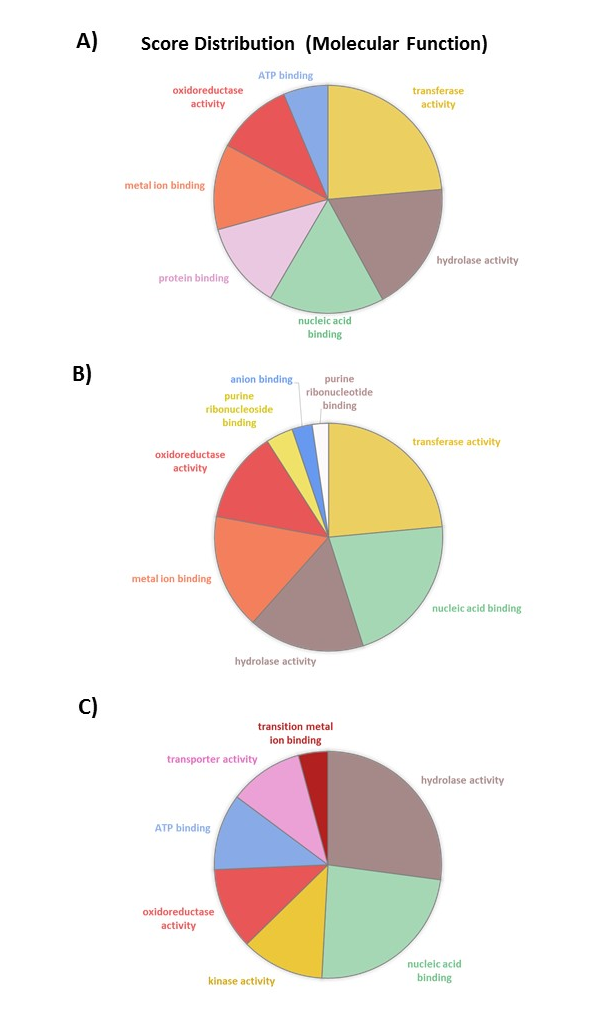


**Supplemental Figure S9:** Pie charts representing the molecular functions which are alliterated in the OT pairwise comparison**.** (A) Overall of the molecular functions associated to up- and down-regulated genes; (B) molecular functions associated to up-regulated genes and (C) molecular functions associated to down-regulated genes.

**Supplemental Table S3** Lists of the significantly differentially expressed genes associated with GO terms which have been obtained from the Venn diagram as a result of the intersection analysis.


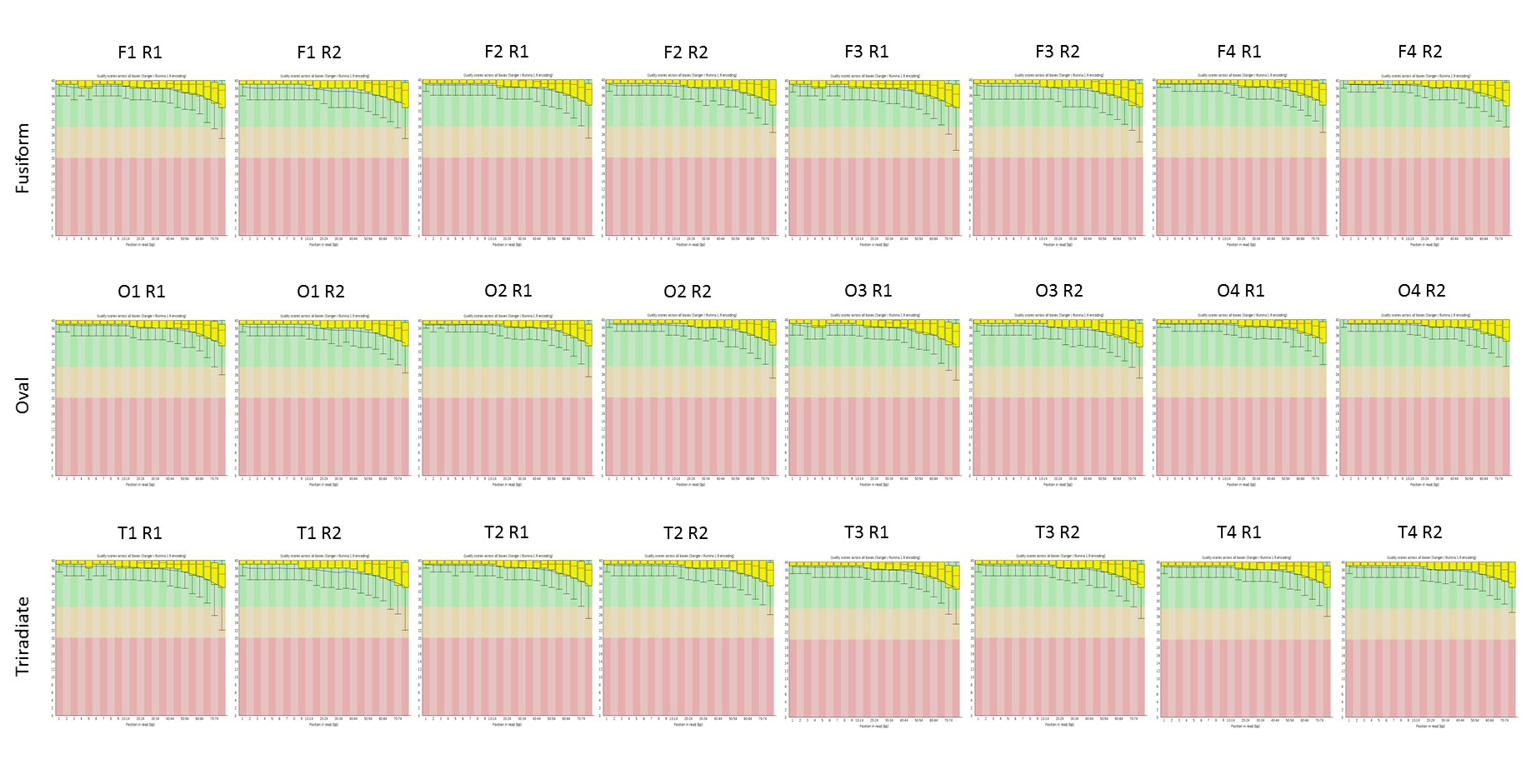


**Supplemental Figure S10:** FastQC report depicting per base sequence quality for the 4 biological replicates of the respective enriched cultures for either the fusiform, triradiate or oval morphotype.

**Supplemental Table S4:** List of the candidate reference genes tested for the normalization of qRT-PCR expression studies in the 3 morphotypes of *Phaeodactylum tricornutum*. The sequences of the primers are given with the amplicon size. The amplification efficiency is given for each primer pair, in each morphotype.
